# Distinct Chemotaxis Protein Paralogs Assemble into Chemoreceptor Signaling Arrays To Coordinate Signaling Output

**DOI:** 10.1128/mBio.01757-19

**Published:** 2019-09-24

**Authors:** Lindsey O’Neal, Jessica M. Gullett, Anastasia Aksenova, Adam Hubler, Ariane Briegel, Davi Ortega, Andreas Kjær, Grant Jensen, Gladys Alexandre

**Affiliations:** a Department of Biochemistry, Cellular & Molecular Biology, The University of Tennessee, Knoxville, Tennessee, USA; b Department of Biology, California Institute of Technology, Pasadena, California, USA; University of Washington

**Keywords:** *Azospirillum*, chemotaxis, chemoreceptor arrays, signaling

## Abstract

Most chemotactic motile bacteria possess multiple chemotaxis signaling systems, the functions of which are not well characterized. Chemotaxis signaling is initiated by chemoreceptors that assemble as large arrays, together with chemotaxis coupling proteins (CheW) and histidine kinase proteins (CheA), which form a baseplate with the cytoplasmic tips of receptors. These cell pole-localized arrays mediate sensing, signaling, and signal amplification during chemotaxis responses. Membrane-bound chemoreceptors with different cytoplasmic domain lengths segregate into distinct arrays. Here, we show that a bacterium, Azospirillum brasilense, which utilizes two chemotaxis signaling systems controlling distinct motility parameters, coordinates its chemotactic responses through the production of two separate membrane-bound chemoreceptor arrays by mixing paralogs within chemotaxis baseplates. The polar localization of chemoreceptors of different length classes is maintained in strains that had baseplate signaling proteins from either chemotaxis system but was lost when both systems were deleted. Chemotaxis proteins (CheA and CheW) from each of the chemotaxis signaling systems (Che1 and Che4) could physically interact with one another, and chemoreceptors from both classes present in *A. brasilense* could interact with Che1 and Che4 proteins. The assembly of paralogs from distinct chemotaxis pathways into baseplates provides a straightforward mechanism for coordinating signaling from distinct pathways, which we predict is not unique to this system given the propensity of chemotaxis systems for horizontal gene transfer.

## Abstract (precis)

The assembly of chemotaxis receptors and signaling proteins into polar arrays is universal in motile chemotactic bacteria. Comparative genome analyses indicate that most motile bacteria possess multiple chemotaxis signaling systems, and experimental evidence suggests that signaling from distinct chemotaxis systems is integrated. Here, we identify one such mechanism. We show that paralogs from two chemotaxis systems assemble together into chemoreceptor arrays, forming baseplates comprised of proteins from both chemotaxis systems. These mixed arrays provide a straightforward mechanism for signal integration and coordinated response output from distinct chemotaxis systems. Given that most chemotactic bacteria encode multiple chemotaxis systems and the propensity for these systems to be laterally transferred, this mechanism may be common to ensure chemotaxis signal integration occurs.

## INTRODUCTION

In bacterial chemotaxis, chemoreceptors sense and propagate environmental signals via a conserved signal transduction cascade. Experimental evidence and mathematical modeling indicate that the stimuli detected by chemoreceptors are greatly amplified at the signaling complex, which indicates that one receptor can interact with multiple histidine kinases (1–3). The signal transduction system that controls bacterial chemotaxis has been best characterized in Escherichia coli (1). E. coli has five different transmembrane chemoreceptors (1, 4–8) that localize to the cell poles along with the kinase CheA and scaffolding CheW proteins to form large patches (9). Cryo-electron microscopy (cryo-EM) and tomography revealed that these polar patches are about 250 nm in diameter and remain mobile within the curvature of the cell pole, while nonpolar patches form at the future division site (9–12). These patches correspond to hexagonally packed trimers of chemoreceptor dimers linked together by rings of interacting CheA and CheW proteins. These large assemblies of transmembrane chemoreceptors in polar arrays are universal features of chemotaxis found in *Bacteria* and *Archaea*. The high degree of cooperativity results from allosteric interactions between chemoreceptors CheA and CheW, and this largely accounts for signal amplification (13).

Comparative analysis of the genome sequences of motile bacteria suggested that most species have more than one chemotaxis pathway in their genome and a greater number of receptors than E. coli (14). Experimental evidence from genetic approaches and cryo-EM analyses also demonstrated that bacteria may express more than one chemoreceptor array segregated into distinct assemblies (11, 15–17). Arrays are only formed between chemoreceptors of the same length class, those containing the same number of heptad (H) repeats in the signaling-terminal region (11, 18). This preference results in distinct arrays, each composed of receptors of a particular length class (11, 18). The tip of chemoreceptors is conserved. This region mediates the stable assembly of chemoreceptors into trimers of dimers that interact with both CheA and CheW to form the structural unit of the chemotaxis array.

**FIG 1:**
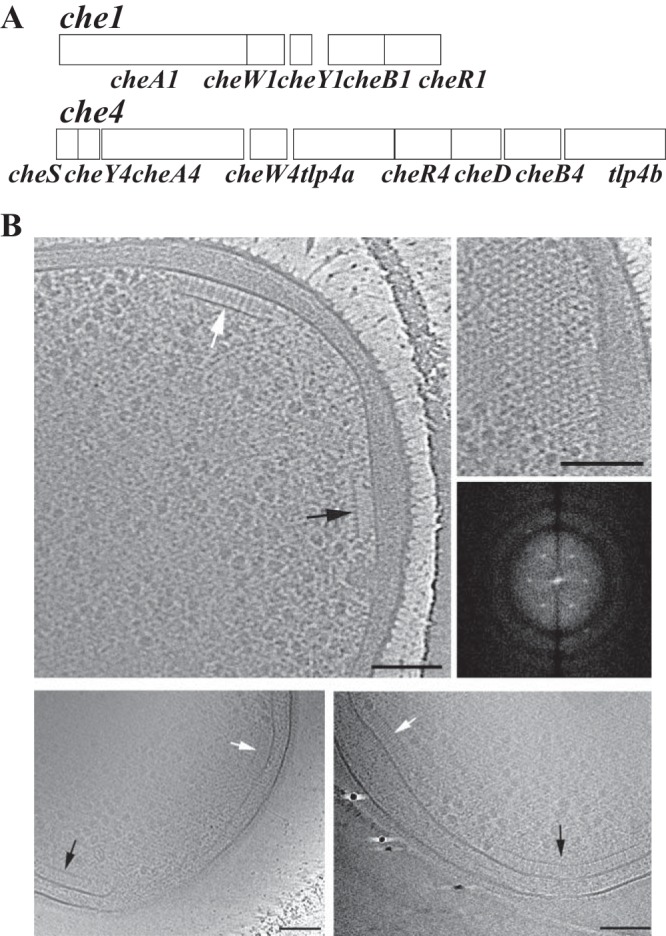
*A. brasilense* has 2 chemotaxis operons and 2 membrane-bound receptor arrays. (A) The topology of Che1 and Che4, the 2 operons encoding the chemotaxis proteins that regulate swimming speed and reversal frequency, respectively. (B) Electron cryotomography of wild-type, *Δche1*, and *Δche4 A. brasilense* strains. (Top left) Slice through a cell showing side views of both long (white arrow) and short (black arrow) arrays in WT cells. (Top right) Top view (scale bar, 100 nm) and power spectrum from top view of the array (not to scale). (Bottom left) Side views of Δ*che1* arrays. (Bottom right) Side views of Δ*che4* arrays.

The genome of the nitrogen-fixing soil bacterium Azospirillum brasilense has four chemotaxis (Che) pathways (19), two of which (Che1 and Che4) are directly implicated in flagellum-driven motility (Fig. 1) (20, 21), while the other two are shown or hypothesized to regulate functions unrelated to chemotaxis (20, 22). Signaling output from Che1 modulates transient changes in swimming speed (23), and the signaling output from Che4 controls transient changes in the probability of swimming reversal (21). Signaling from both Che1 and Che4 is required to produce a chemotaxis response. Therefore, *A. brasilense* chemotaxis response depends on coordination of the signaling outputs from both Che1 and Che4 (21, 24, 25), with experimental evidence suggesting this is mediated at the level of chemoreceptor activity (25, 26). Here, we characterize the spatial organization of two chemoreceptor arrays in *A. brasilense.* We provide evidence that Che1 and Che4 chemotaxis proteins can interact with receptors in both arrays, which provides a mechanism for the integration of signaling from Che1 and Che4.

## RESULTS

### Cryo-EM reveals two distinct chemoreceptor arrays in *A. brasilense*.

The *A. brasilense* genome encodes 51 predicted chemoreceptors from at least four different length classes (18), but the organization of the chemoreceptors contributing to flagellum-mediated chemotaxis has not been characterized. We used cryo-electron tomography (cryo-EM) to characterize the chemoreceptor arrays formed by interaction with Che1 or Che4 signaling proteins. Because chemoreceptor arrays do not assemble as organized clusters in cells lacking CheA or CheW in E. coli (27, 28), we hypothesized that we could identify Che1- and Che4-associated arrays in *A. brasilense* using a combination of the wild-type (WT) strain and Δ*che1*, Δ*che4*, and Δ*che1* Δ*che4* mutant derivatives. We were able to observe two spatially distinct chemoreceptor arrays in wild-type, *Δche1*, and *Δche4* cells (Fig. 1B). These arrays also had different heights, measured from the inner membrane to the CheA-CheW base layer, supporting the fact that they are distinct arrays. In contrast, no array could be observed in the Δ*che1* Δ*che4* mutant (20 cells imaged). Top views of both of the chemoreceptor arrays (Fig. 1B) revealed the typical, highly ordered hexagonal packing with a 12-nm spacing between the neighboring hexagons. *A. brasilense* carries 2 additional chemosensory operons, Che2 and Che3, but at this point, we have not observed clusters that we can assign to the remaining Che operons. These results indicate that there are two distinct membrane-bound arrays that can form when proteins are from Che1 or Che4.

### Polar localization of chemotaxis pathway proteins depends on the presence of Che1 and Che4 proteins.

**FIG 2:**
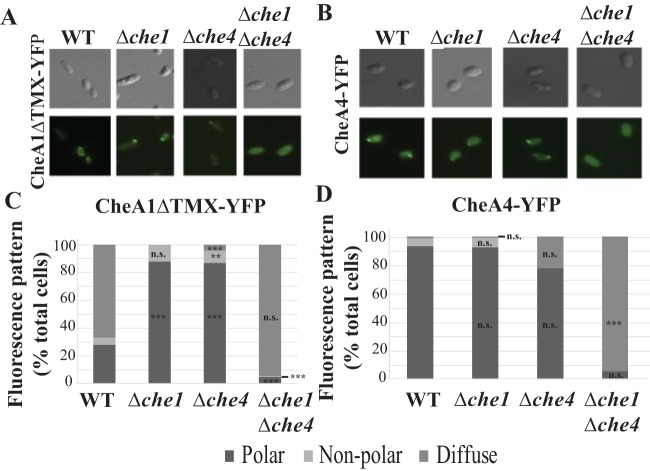
Localization of YFP-tagged CheA proteins. CheA1ΔTMX-YFP (A) and CheA4-YFP (B) cells in wild-type (WT) and Δ*che1*, Δ*che4*, and Δ*che1* Δ*che4* mutant derivative backgrounds. (C) Quantification of the distribution of fluorescence in the population of CheA1ΔTMX-YFP cells analyzed in wild-type and mutant derivative backgrounds. For each bar, *n* ≥ 50 cells. (D) Quantitation of the distribution of fluorescence in the population of CheA4-YFP cells analyzed in wild-type and mutant derivative backgrounds. Z tests were used to determine if fluorescent focus localization differed significantly from that of the WT in *Δche1*, *Δche4*, and *Δche1 Δche4* strains. *P* < 0.05 (*), *P* < 0.01 (**), or *P* < 0.001 (***).

The results of the cryo-EM analyses suggested that Che1 and Che4 proteins were important in formation of both arrays. We analyzed the dependence of chemoreceptor cluster localization on Che1 and Che4 proteins to localize using yellow fluorescent protein (YFP) fusions of Che1 and Che4 chemotaxis protein paralogs (CheA, CheW, and CheY) and imaged each in wild-type and Δ*che1*, Δ*che4*, and Δ*che1* Δ*che4* mutant backgrounds. The functionality of all tagged proteins was verified through chemotaxis soft agar assays before imaging (29). *A. brasilense* Sp7 contains two isoforms of CheA1: full-length CheA1 (which is membrane anchored, localizes at the cell surface, and is dispensable for chemotaxis) and CheA1ΔTMX (which lacks the transmembrane domains and is vital to chemotaxis) (29). We used CheA1ΔTMX-YFP to assess chemotaxis-dependent localization. In the wild-type background, CheA1ΔTMX-YFP localized as bright foci to the cell poles and was also diffuse and throughout the cell surface (Fig. 2A and C). This surface localization is probably due to CheA1ΔTMX interacting with its full-length CheA1 isoform, which localizes to the cell surface. In the Δ*che1* and Δ*che4* backgrounds, CheA1ΔTMX-YFP localized to the cell poles (Fig. 2A and C). In the Δ*che1* Δ*che4* background, fluorescence was diffuse and no distinct fluorescent foci were detected (Fig. 2A and C). These results suggest that CheA1ΔTMX-YFP localizes to the cell pole with Che1 or Che4 proteins, suggesting that it can interact with either system. The ability of CheA1ΔTMX-YFP to localize mainly as polar foci in the Δ*che1* background further indicates that the natively produced CheA1ΔTMX is part of the Che4 cluster.

CheA4-YFP localized as polar foci in the wild-type, Δ*che1*, and Δ*che4* mutant strains and was diffuse in the Δ*che1* Δ*che4* strain background (Fig. 2B and D). YFP fusions to CheY4 (CheY4-YFP), which functions downstream of CheA4 (21), and to the adaptor proteins CheW1 and CheW4, which are encoded by the Che1 and Che4 pathways, respectively (CheW1-YFP and CheW4-YFP), also localized as polar foci in wild-type, Δ*che1*, and Δ*che4* backgrounds but were diffuse in the Δ*che1* Δ*che4* mutant background (see Fig. S1A to C in the supplemental material). A CheY1-YFP fusion is diffuse, since CheY1-YFP interacts with polarly localized CheA1ΔTMX and membrane-bound CheA1 (Fig. S2D). These results suggest that Che1 and Che4 proteins can localize to the cell pole as long as either Che1 or Che4 proteins are present.

10.1128/mBio.01757-19.1 FIG S1 Subcellular localization of fluorescently tagged chemotaxis proteins from Che1 and Che4. (A) Quantification of the distribution of fluorescence in the population of CheW4-YFP cells analyzed in wild-type (WT) and mutant derivative backgrounds. *N* ≥ 60 cells for each bar. (B) Quantification of the distribution of fluorescence in the population of CheW1-YFP cells analyzed in wild-type (WT) and mutant derivative backgrounds. *N* ≥ 73 cells for each bar. (C) Quantification of the distribution of fluorescence in the population of CheY4-YFP cells analyzed in wild-type (WT) and mutant derivative backgrounds. *N* ≥ 100 cells for each bar. (D) Quantification of the distribution of fluorescence in the population of CheY1-YFP cells analyzed in wild-type (WT) and mutant derivative backgrounds. *N* ≥ 60 cells for each bar. Download FIG S1, EPS file, 1.3 MB. Copyright © 2019 O’Neal et al. 2019 O’Neal et al. This content is distributed under the terms of the Creative Commons Attribution 4.0 International license.

10.1128/mBio.01757-19.2 FIG S2 Involvement of CheA2 and CheA3 in chemotaxis and array formation. Cells lacking CheA2 are impaired in swimming in a soft-agar assay. (A) Localization of chemotaxis proteins in *cheA2*::*Tet cheA3*::Tn*5* background. YFP-tagged CheA1ΔTMX, CheA4, Tlp1, and Tlp4a all are able to polarly localize in the absence of CheA2 and CheA3. (B and C) Localization of CheA2-CFP and CheA3-CFP when grown in liquid or on solid medium. Download FIG S2, PDF file, 2.1 MB. Copyright © 2019 O’Neal et al. 2019 O’Neal et al. This content is distributed under the terms of the Creative Commons Attribution 4.0 International license.

To determine if Che2 and Che3 were important in polar array formation, we also looked at localization of CheA1ΔTMX-YFP and CheA4-YFP in cells lacking CheA2 and CheA3. Localization of CheA1ΔTMX-YFP and CheA4-YFP in cells lacking CheA2 and CheA3 was similar to that of the wild-type strain, indicating these proteins are not required for polar focus formation (Fig. S2B and C). Furthermore, we did not observe any visible fluorescent foci for CheA2-YFP and CheA3-YFP in free-swimming cells (Fig. S2D) and only detected diffuse fluorescence of CheA2-YFP and CheA3-YFP in cells grown on plates. This suggests that CheA2-YFP and CheA3-YFP do not assemble into visible fluorescent foci and are distributed throughout the cells under conditions of limited swimming. The findings that proper localization of Che1 and Che4 chemotaxis proteins depends on the presence of either Che1 or Che4 proteins suggests Che1 and Che4 proteins interact and form polarly localized arrays.

### Che1 and Che4 pathway proteins interact with each other in the bacterial two-hybrid assay.

**FIG 3:**
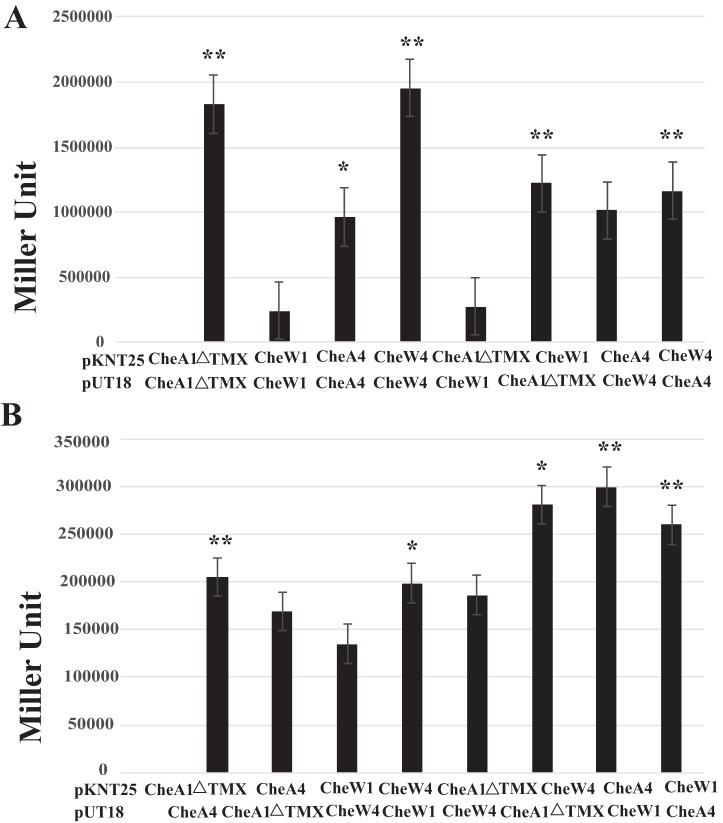
Interaction of chemotaxis proteins from Che1 and Che4 in the bacterial two-hybrid assay. (A) Interactions between chemotaxis proteins encoded by the same operon. (B) Interactions between proteins encoded by different chemotaxis operons. All protein interactions were tested bidirectionally, since the vectors had different copy numbers (pKNT25, low copy; pUT18, high copy). Significance for each interaction is relative to the negative control run alongside it on the 96-well plate. *, *P* <0.05; **, *P* < 0.005.

To test whether Che1 and Che4 proteins physically interact, which would be required to form stable chemoreceptor arrays, we used a bacterial adenylate cyclase two-hybrid (BACTH) assay. Given that YFP fusions to the C terminus of CheA1ΔTMX, CheA4, CheW1, CheW4, CheY1, and CheY4 are functional (26, 29), we tested all interactions with the catalytic domains fused at the C terminus of the proteins expressed from both BACTH vectors. We first determined that proteins encoded by the same operon are able to interact. CheA4 and CheW4 interacted with one another in this system, as expected, since both proteins are encoded by genes found together within the *che4* operon, and previous work indicates they function in the same pathway (21) (Fig. 3 and Table 1). CheA4 and CheW4 were also able to interact with themselves. We also detected positive interactions between proteins encoded by the Che1 operon, with CheA1ΔTMX interacting with itself and with CheW1, as expected. Proteins encoded by separate operons were able to interact with each other: CheA1ΔTMX with CheA4, CheA1ΔTMX with CheW4, and CheA4 with CheW1 (Fig. 3 and Table 1). The BACTH vectors have different copy numbers, which could explain the varied strength of the interactions detected. These results concur with our microscopy observations and indicate that, in this assay, proteins from Che1 could physically interact with proteins from Che4. We tested these chemotaxis proteins against empty vectors and found no interaction, consistent with the absence of reports of autoactivation in this assay (30). False positives are rarely, if ever, reported with the BACTH assay (30), suggesting that positive interactions detected here are reliable.

**TABLE 1:** Summary of Che1 and Che4 protein interactions in the BACTH assay, as determined by β-galactosidase assay

pUT18	pKNT25^*a*^					
	CheA1ΔTMX	CheA4	CheW1	CheW4	Tlp1	Tlp4a
CheA1ΔTMX	++	NS^*b*^	++	+	NS	NS
CheA4	++	+	++	++	++	NS
CheW1	NS	++	NS	+	+	NS
CheW4	NS	NS	NS	++	+	NS
Tlp1	+	NS	NS	NS	++	NS
Tlp4a	+	+	NS	+	NS	NS

a +, ++, and +++ indicate positive interactions that were significantly different from values for negative controls at *P* values of ≤0.05, ≤0.01, and ≤0.001, respectively.

b NS, the beta-galactosidase activity was not significantly different from that of the negative control.

### Chemoreceptors of different length classes comprise the two distinct chemoreceptor arrays.

To further explore the possible interaction of Che1 and Che4 proteins within each of the two membrane-anchored arrays, we next determined the receptor classes composing each of the membrane-bound arrays in WT, *Δche1*, and *Δche4* cells. Chemoreceptors present in an array can be predicted from their protein sequence, specifically the heptad class of the signaling domain (11, 18). Previous work has used gene order and neighborhoods, domain architecture of chemotaxis proteins, and signaling domain classes to predict interactions between chemotaxis system classes (ACF, TFP, and F1 to F15) and chemoreceptor heptad classes (14, 18, 31). Using this classification scheme, the Che1 operon, which is classified as an F5, is likely to interact with 38H receptors, while the Che4 operon, an F7 system, is predicted to interact with 36H (14, 18, 31). Both 36H and 38H receptors are encoded by the *A. brasilense* genome.

The height of chemoreceptor arrays is determined by the physical length of membrane-bound chemoreceptor proteins present in the array. Previous studies in Magnetospirillum magneticum have shown that 38H chemoreceptors with a single HAMP domain and alpha helical linker form chemotaxis arrays that are 28 nm tall in cryo-EM images (11). Multiple-sequence alignments of 38H chemoreceptors from both *A. brasilense* and *M. magneticum* in the region between the transmembrane and the signaling domain do not contain any gap, suggesting that they share the same domain architecture and the same physical height (Fig. S3A). Two of the *A. brasilense* 38H chemoreceptors have an extra HAMP domain instead of the alpha helix linker (AMK58_RS04445 and AMK58_RS08090) (Fig. S3B). These chemoreceptors are predicted to be of the same physical height, at ∼27.5 nm, based on (i) secondary structure comparison against one of the chemoreceptors with a HAMP domain and alpha helix linker discussed above and (ii) the crystal structures of the triple HAMP domains (PDB entry 3LNR) for estimating the physical height of three HAMP domains (32) (Fig. S3C). Therefore, the *A. brasilense* 38H chemoreceptors are predicted to form arrays of ∼28 nm in length.

10.1128/mBio.01757-19.3 FIG S3 Comparison of the cytoplasmic portion between the TM region and the MCP signal domain of 38H receptors from *A. brasilense* and *M. magneticum* shows that both types of domain architecture should fold a chemoreceptor of the same height. (A and B) Domain architecture, alignment, and secondary structure prediction (red) for chemoreceptors with double HAMP (A) and single HAMP domain with alpha helix linker (B). (C) Mapping of the domain architectures of 38H receptors with double HAMP (left) and single HAMP with linker (right) onto the second and third HAMP of the homologous structure of the triple HAMP in P. aeruginosa (PDB entry 3LNR) shows that both arrangements have the same physical height. Download FIG S3, TIF file, 2.1 MB. Copyright © 2019 O’Neal et al. 2019 O’Neal et al. This content is distributed under the terms of the Creative Commons Attribution 4.0 International license.

The *A. brasilense* genome also contains three 36H chemoreceptors, all of which have 3 HAMP domains, including Tlp4a, which is encoded by the *che4* operon. Tlp4a also possesses an additional PAS domain between the first and second HAMP domains. The domain architecture of the signaling region of Tlp4a is similar to that of the Aer2-like chemoreceptor found in Methylomicrobium alcaliphilum (MEALZ_2872) (Fig. S4) and for which a homology model is available (33). Using the cytoplasmic region of the *M. alcalyphilum* Aer-2-like as a template (Fig. S4), we built a homology model of the cytoplasmic region of Tlp4a and determined that arrays formed by Tlp4a should have a physical height from the membrane of approximately 30 nm. The other two 36H chemoreceptors predicted in the *A. brasilense* genome contain a 23-residue linker instead of the PAS domain of Tlp4a (see Data set S2 at https://doi.org/10.6084/m9.figshare.9714056). This linker is predicted to be alpha helical and to have a length of ∼3.4 nm, exactly matching the predicted size of the PAS domain of Tlp4a. Therefore, 36H chemoreceptors from the *A. brasilense* genome are expected to segregate into a spatially distinct array of ∼30 nm in length.

10.1128/mBio.01757-19.4 FIG S4 Predicting the height of chemoreceptor arrays made by Tlp4a. (A) Domain architecture, sequence alignment, and secondary structure prediction (red) of the Tlp4a and the Aer2-like Methylomicrobium alcaliphilum MEALZ_2872. (B) Homology model of Tlp4a predicts that these receptors form 30-nm arrays. Download FIG S4, TIF file, 1.6 MB. Copyright © 2019 O’Neal et al. 2019 O’Neal et al. This content is distributed under the terms of the Creative Commons Attribution 4.0 International license.

We next analyzed tomograms obtained from the WT, *Δche1*, and *Δche4* cells to precisely measure the height of the chemoreceptor arrays. We observed polar arrays in 15 wild-type cells, 24 Δ*che1* cells, and 18 Δ*che4* cells (Table S3). An accurate measurement (within about 3 nm uncertainty; see Materials and Methods for details) of chemoreceptor heights could be performed on only a subset of tomograms (Fig. S5). In wild-type cells, we accurately measured the height for 6 short chemoreceptor arrays (27.5 ± 2.6 nm on average, with uncertainty measured as the plus/minus value, as described in Materials and Methods) and 2 tall arrays (31 ± 2.4 nm on average), as measured by the distance between the inner membrane and the CheA/CheW base plate (Fig. 1B). In Δ*che1* cell poles, all chemoreceptor arrays were short, measuring 28.1 ± 3.1 nm on average in height. In *Δche4* cells, 11 cell poles contained short arrays averaging 27.7 ± 3.1 nm and 3 tall arrays averaging 31.3 ± 2.8 nm. The measured height of receptor arrays in the wild-type strain were consistent with the predicted 38H and 36H chemoreceptor heights, at ∼28 nm and ∼30 nm. We also note that the height difference between the tall and short arrays is around 3 nm, which is consistent with theoretical predictions of the chemoreceptor heights but also right at the uncertainty limit where we could accurately measure array height (Fig. S5). Based on the presence of spatially distinct chemoreceptor polar arrays in the wild-type and the Δ*che4* strains, we predict that chemoreceptors of different length classes segregate in spatially distinct arrays (11, 12, 15, 18). The previous experimental demonstration that an insertion as small as 14 amino acids in a chemoreceptor signaling domain (∼2 nm height difference for an alpha-helical structure) is sufficient to spatially segregate chemoreceptors in distinct arrays (15) is also consistent with 36H and 38H chemoreceptors comprising each of the two spatial arrays observed in *A. brasilense*. Short and tall arrays are observed only in the WT or strains lacking the Che4 pathway (*Δche4* background), implying that both 38H chemoreceptors and 36H chemoreceptors interact with both Che1 and Che4 paralogs to form arrays with different heights in these strain backgrounds. In the *Δche1* background only short arrays were formed, implying that 38H receptors, predicted to interact with Che1 proteins, also interact with Che4. These data are supporting evidence that Che1 and Che4 protein paralogs are involved in assembly of both arrays.

10.1128/mBio.01757-19.5 FIG S5 Subset of chemoreceptor array height with measurement accuracy in *A. brasilense* wild type (WT) and its Δ*che1* and Δ*che4* mutant derivatives. The distribution of chemoreceptor array height measured on tomograms is shown, with the error bars representing the standard uncertainty in each measurement. Download FIG S5, PDF file, 0.04 MB. Copyright © 2019 O’Neal et al. 2019 O’Neal et al. This content is distributed under the terms of the Creative Commons Attribution 4.0 International license.

10.1128/mBio.01757-19.10 TABLE S3 Summary of measured height of chemoreceptor arrays. Download Table S3, DOCX file, 0.02 MB. Copyright © 2019 O’Neal et al. 2019 O’Neal et al. This content is distributed under the terms of the Creative Commons Attribution 4.0 International license.

### The discrete subcellular localization of 36H and 38H chemoreceptors depends on CheA1 and CheA4.

**FIG 4:**
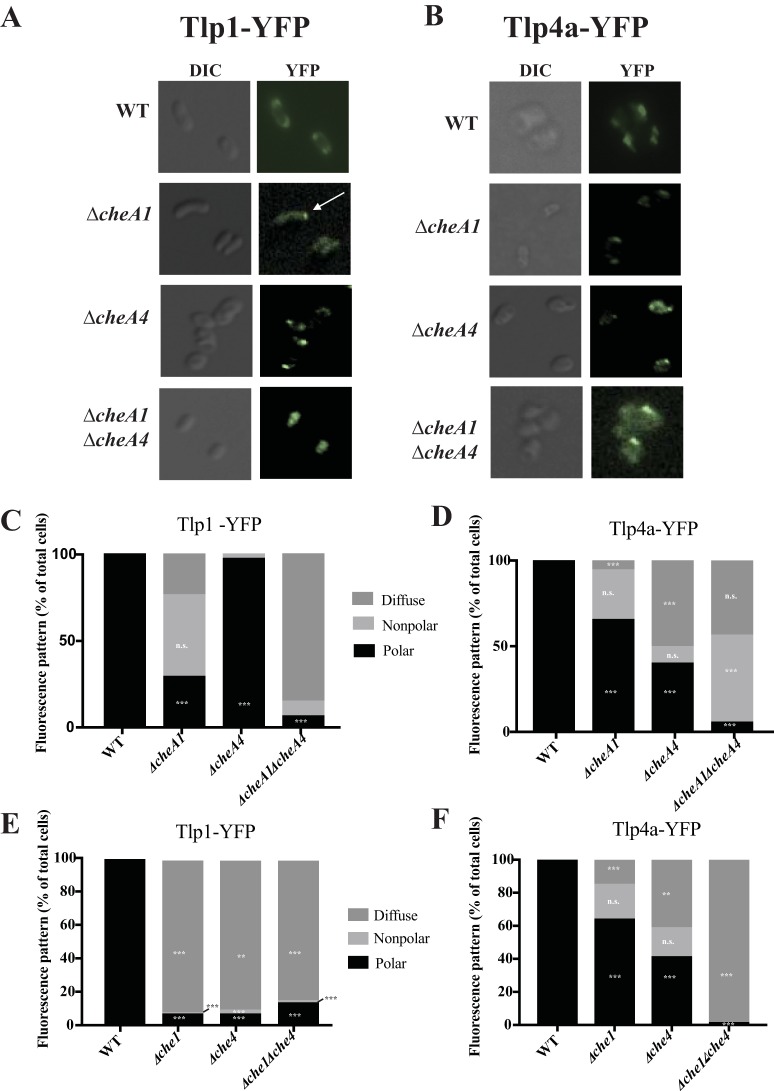
Role of chemotaxis proteins in the localization of YFP-tagged chemoreceptors. (A and B) Differential interference contrast (DIC) and fluorescence microscopy images of Tlp1-YFP (A) and Tlp4a-YFP (B) in the wild-type (WT) and Δ*cheA1*, Δ*cheA4*, and Δ*cheA1* Δ*cheA4* mutant backgrounds. Relative polar focus fluorescence intensity with standard deviations is listed beneath the corresponding YFP image. (C to F) Percentage of cells with polar, nonpolar, and diffuse localization of Tlp1-YFP (C and E) and Tlp4a-YFP (D and F) clusters in cells lacking CheA1, CheA4, and CheA1 CheA4 proteins (C and D) and cells lacking all Che1, Che4, and Che1 Che4 proteins (E and F). For each bar, *n* ≥ 80 cells. Z tests were used to determine if fluorescent focus localization differed significantly from that of the WT in *Δche1*, *Δche4*, and *Δche1 Δche4* strains. *P* < 0.05 (*), *P* < 0.01 (**), or *P* < 0.001 (***).

Results described above led us to a model for chemoreceptor array assembly in *A. brasilense* in which chemoreceptors of the 36H and 38H classes form distinct arrays that each assemble with Che1 and Che4 pathway proteins. To test this model, we fluorescently tagged a 38H (Tlp1) and a 36H (Tlp4a) chemoreceptor and analyzed their subcellular localization in the wild-type, *ΔcheA1*, *ΔcheA4*, and *ΔcheA1 ΔcheA4* backgrounds. Both Tlp1-YFP and Tlp4a-YFP, which are functional fusions, localized at the cell poles as punctate foci when expressed in the wild-type strain (Fig. 4). In the Δ*cheA1* strain, Tlp1-YFP was diffuse throughout the cell, with a small fraction of cells (29%) showing polar foci (Fig. 4C and E). Tlp1-YFP localized to the cell poles in the Δ*cheA4* strain in a pattern similar to that seen in the wild-type strain background. Tlp1-YFP was diffuse in the Δ*cheA1* Δ*cheA4* strain, and fluorescent foci were seldom observed. Thus, CheA1 plays a major role in polar localization of Tlp1-YFP while CheA4 plays a minor, if any, role. In strains lacking all Che1 or Che4 proteins, Tlp1-YFP was mostly diffuse (Fig. 4F), suggesting that other Che1 and Che4 chemotaxis proteins are required for Tlp1-YFP localization.

In the Δ*cheA1* strain background, Tlp4a-YFP localizes as numerous foci, both lateral and polar, that also appeared smaller in size relative to the large polar foci of the wild type. The average number of fluorescent foci per cell in the wild-type background was 2.1 ± 0.7, while the number of fluorescent foci per cell in the Δ*cheA1* strain was 3.8 ± 1.3 (*P* < 0.0001 by Student's *t* test, *N* = 90). In the Δ*cheA4* strain, the Tlp4a-YFP fluorescent foci were mislocalized; foci often were slightly off-polar and detected at a greater distance from the cell poles (Fig. 4D), while in the Δ*cheA1* Δ*cheA4* strain Tlp4a-YFP was mostly found as either nonpolar foci or was diffuse (Fig. 4D). Together, these observations suggest that polar localization of Tlp4a-YFP in clusters seen as tight fluorescent foci requires both CheA1 and CheA4. In strains lacking all Che1 or Che4 proteins, Tlp4a-YFP localization produced patterns similar to the ones observed for strains lacking CheA1 or CheA4, including the loss of focus formation in the absence of both sets of chemotaxis proteins (Fig. 4E), which suggests that Tlp4a-YFP polar localization depends upon proteins encoded by both Che1 and Che4.

### 36H and 38H chemoreceptors physically interact with Che1 and Che4 pathway proteins.

We next tested for possible physical interaction of CheA1, CheW1, CheA4, and CheW4 with Tlp1 and Tlp4a in the BACTH assay. Tlp1 positively interacted with CheA1ΔTMX, CheW1, and itself (Fig. S6). This finding was expected, given that chemoreceptors are known to dimerize and that Tlp1 was shown to signal in a Che1-dependent manner (26). Tlp1 also positively interacted with CheA4 and CheW4 (Table 1, Fig. S6). Tlp4a interacted with CheA4 and CheW4, as expected, but also interacted with CheA1ΔTMX (Fig. 3B). Full-length Tlp4a was not found to interact with itself at detectable levels in this assay. However, given that Tlp4a had positive interactions with other proteins, it is unlikely that this is the result of a nonfunctional tagged protein, but the reason for this negative result is not known.

10.1128/mBio.01757-19.6 FIG S6 Interaction of chemotaxis proteins from Che1 and Che4 with Tlp1 (A) and Tlp4a (B) in the bacterial two-hybrid (BACTH) assay. All protein-protein interactions were tested bidirectionally, since the vectors had different copy numbers (pKNT25, low copy; pUT18, high copy). Significance for each interaction is relative to that of the negative control run alongside it on its individual 96-well plate. *, *P* < 0.05; **, *P* < 0.005. Download FIG S6, EPS file, 1.1 MB. Copyright © 2019 O’Neal et al. 2019 O’Neal et al. This content is distributed under the terms of the Creative Commons Attribution 4.0 International license.

**FIG 5:**
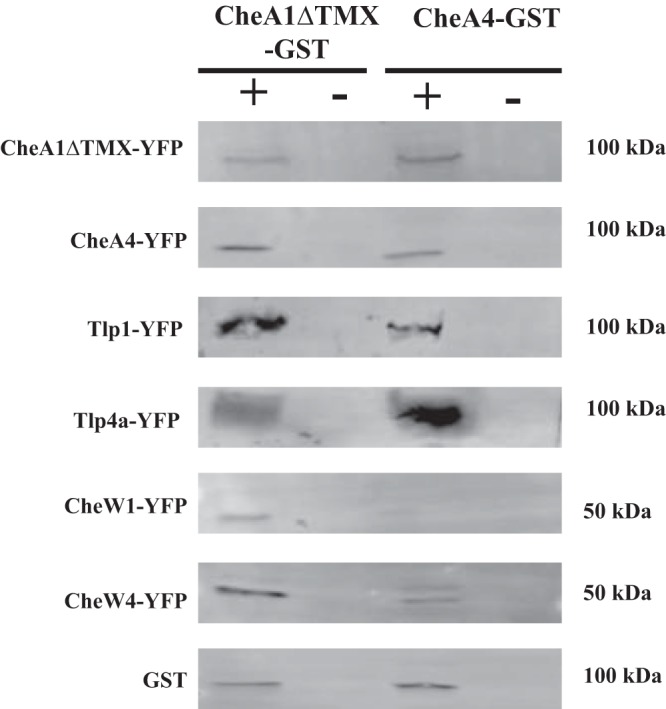
Interaction of chemotaxis proteins in pulldown assays. C-terminally GST-tagged CheA1ΔTMX and CheA4 were immobilized on an anti-GST column. Cell lysates containing YFP-tagged CheAΔTMX, CheA4, Tlp1, Tlp4a, CheW1, and CheW4 were flowed over columns containing GST-tagged CheA1ΔTMX and CheA4 (+) or GST (−). Unbound proteins were washed using 1× PBS, and bound proteins were eluted with 10 mM glutathione. Proteins were detected by Western blotting using rabbit anti-GFP (Abcam) and rabbit anti-GST (Invitrogen).

As expected from their different lengths, Tlp1 and Tlp4a did not interact. Together, these data suggest that chemotaxis receptors from the 38H (Tlp1) and 36H (Tlp4a) classes can physically interact with Che1 and some of the Che4 proteins, including both CheA1 and CheA4. These results are fully consistent with the fluorescence imaging data described above. We next used pulldown assays to verify the physical interactions suggested by the BACTH assay (Fig. 5). We found that CheA1ΔTMX-GST could interact with itself as well as with CheA4-YFP, Tlp1-YFP, and Tlp4a-YFP (Fig. 5A and C). CheA1ΔTMX-GST interacted with CheW1-YFP. This finding is expected, since CheA1 and CheW1 are encoded by the *che1* operon. CheW interacts with CheA through the P5 domain (34, 35), and the *A. brasilense* CheA1 possesses two P5 domains at its C terminus, which could complicate the interactions tested here. In a similar assay, we found that CheA4-GST interacts with CheA1ΔTMX-YFP, CheW1-YFP, CheW4-YFP, Tlp4a-YFP, and Tlp1-YFP (Fig. 5). Together, these data validate the physical interactions between Che1 and Che4 chemotaxis proteins, including their interaction to form mixed chemotaxis signaling clusters.

## DISCUSSION

Our results indicate that in *A. brasilense*, each of the two membrane-bound chemoreceptor arrays detected by cryo-EM utilize paralogs from both Che1 and Che4 chemotaxis signaling pathways to build extended stable arrays. Our experimental evidence strongly suggests that CheA1ΔTMX and CheA4, and likely CheW1 and CheW4, are recruited to form the baseplates of each of the two *A. brasilense* chemotaxis receptor arrays, each of which likely comprise Tlp1 and other 38H receptors or Tlp4a and other 36H receptors. First, cryo-EM data indicate that *A. brasilense* contains 2 chemotaxis arrays, even in the absence of Che1 or Che4 proteins. This suggests that Che1 and Che4 proteins comprise both arrays. Second, fluorescence imaging data indicate that Che1 and Che4 proteins properly localize as long as either the complete Che1 or Che4 system is present. Third, chemotaxis proteins encoded by separate operons are capable of interacting with each other in a BACTH assay, and these interactions are also detected in pulldown experiments, indicating they occur *in vivo*. Fourth, chemoreceptors suggested by sequence analysis to represent each of the two arrays detected by cryo-EM, Tlp1 and Tlp4a, interact with both CheA1ΔTMX and CheA4 in the BACTH assay and a pulldown assay. These results together support a model in which proteins from both Che1 and Che4 mix in chemoreceptor array baseplates. These results also provide a straightforward mechanism by which two chemotaxis signaling pathways, Che1 and Che4, could function in a coordinated manner to regulate chemotaxis responses in *A. brasilense*, despite each of Che1 and Che4 regulating different signaling outputs (21, 23). In support of this hypothesis, we have previously shown that mutations within Che1 affect reversal frequency, which is the direct signaling output of Che4 (23), while mutations within Che4 also affect swimming speed, the direct output of Che1 (21). Additional evidence points to indirect interactions between chemotaxis signaling pathways at the level of chemoreceptors (25, 26). In addition, we have previously shown that Tlp1, a 38H chemoreceptor, signals through both Che1 and Che4 pathways (26, 36), despite predictions that the 38H chemoreceptors interact with Che1 (19). Tlp4a is a 36H chemoreceptor encoded by the Che4 operon that is predicted to function with Che4 proteins (21). Our cryo-EM data suggest that Tlp4a and the other 36H chemoreceptors form tall arrays that also depend on the presence of Che1 proteins. We do not know the sensory function of Tlp4a or of any of the other 36H chemoreceptors and, thus, have not experimentally determined if it could signal in a Che1-dependent manner. While data obtained here support the proposed model for 36H chemoreceptors forming array signaling in a Che1- and Che4-dependent manner, it remains to be experimentally validated. Given the propensity for chemotaxis signal pathways to be horizontally transferred between bacteria (14), a similar cobinding and mixing mechanism may be found in other bacteria.

Our data suggest that paralogs from Che1 and Che4 can physically interact, which would be necessary to form mixed baseplates. CheA dimerization is facilitated through the P3 domain. CheA1 P3 and CheA4 P3 have 59% sequence similarity, which perhaps is sufficient to license dimerization by intersubunit P3/P3 exchange. CheA paralogs possess P5 domains that interact with CheW, and the interaction interface is critical for stabilizing chemoreceptor arrays and maintaining connectivity and signal cooperativity (34–36). We detected positive interactions between CheA1ΔTMX and CheA4 with both CheW1 and CheW4. CheA1 contains two P5 domains (P5A and P5B), while CheA4 has a single P5 domain. Previous work in E. coli identified conserved residues necessary for CheA P5-CheW interactions (35). CheA1 P5A contains 6 of the 16 conserved residues in E. coli CheA implicated in interaction with CheW, while CheA1 P5B had only 3 of these 16 residues. CheA4 P5 contains 5 of the conserved residues in E. coli CheA (see Fig. S7 in the supplemental material). Furthermore, while CheA1 P5A and P5B domains are most similar to one another (51%), CheA1 P5A, and not P5B, shares the most similarity with CheA4 P5 (47%). CheW1 and CheW4 share 50% sequence similarity with one another, and both CheW1 and CheW4 possess all the conserved residues implicated in CheA-CheW binding in E. coli (Fig. S7) (37). Given the role of CheA and CheW interactions with each other and receptors in cluster formation and the ability of P5-CheW proteins to substitute for one another in chemoreceptor arrays (12, 38–40), similar interactions between paralogous chemotaxis proteins may produce mixed clusters in *A. brasilense*.

10.1128/mBio.01757-19.7 FIG S7 Sequence alignment between *A. brasilense* chemotaxis paralogs and E. coli homologs. (A) P5 domains of *A. brasilense* CheA1 and CheA4 and E. coli CheA. Highlighted residues are conserved residues necessary for interaction with CheW in E. coli. (B) Alignment of *A. brasilense* CheW1 and CheW4 with E. coli CheW. Highlighted residues are essential for CheA-CheW interaction in E. coli. *, residue at this position is fully conserved; :, residues are highly similar; ., residues are weakly similar. Download FIG S7, DOCX file, 0.01 MB. Copyright © 2019 O’Neal et al. 2019 O’Neal et al. This content is distributed under the terms of the Creative Commons Attribution 4.0 International license.

The ability for different chemotaxis proteins to assemble in mixed baseplates within a single array is also suggested to occur via domains related to CheW in other model systems. CheV proteins, which structurally and functionally are CheW-REC hybrids (41), are widespread in bacterial genomes, and recent evidence indicates that in Helicobacter pylori, CheV-comprising chemoreceptor-kinase clusters can join a larger CheW-receptor-kinase membrane-anchored cluster. In Salmonella enterica, recent comparative genomics combined with analysis of experimental evidence also imply a role for CheV in bringing specific chemoreceptors to stable arrays (42). In Vibrio cholerae, baseplate composition is dynamic, with CheW and CheV able to form baseplates in the absence of CheA (43). This variation is hypothesized to aid array function and receptor turnover. Thus, *A. brasilense* Che1 and Che4 proteins forming mixed baseplates is not incompatible with maintaining signal cooperativity within the chemoreceptor arrays. Physical separation of chemoreceptors into two distinct membrane-bound receptor arrays is dictated by segregation of chemoreceptors into length classes (15). This observation raises questions on the relative contributions of the different chemoreceptor arrays to sensing.

Signal processing and integration via multiple chemotaxis signaling pathways is unlikely to be unique to *A. brasilense* given the number of bacterial genomes with multiple chemotaxis pathways (14) and experimental evidence of signal integration between chemotaxis signaling pathways in other species (44). Evidence of signal integration implicating chemotaxis receptors and multiple signaling pathways exists in Myxococcus xanthus between the Che7, Dif, and Frz chemotaxis-like signaling pathways (45–47) and Comamonas testosteroni between the Che and Flm systems (48). In contrast, spatial segregation of chemotaxis signaling is required to prevent cross talk in other species, including Rhodobacter sphaeroides. In this species, two functional chemotaxis clusters are found within the cells: a polar membrane-anchored cluster and a cytoplasmic one (49–51). These clusters are physically and spatially separated from each other, and each of the chemotaxis proteins specifically assembles in a single chemotaxis signaling cluster, with experimental evidence suggesting this organization prevents detrimental signaling cross talk (49–52). The subcellular organization of chemotaxis signaling clusters in R. sphaeroides suggests stringent specificity in the interactions between chemoreceptors and baseplate proteins CheA and CheW, which would be relaxed in the chemoreceptor-CheA-CheW clustering in *A. brasilense*.

Despite recent examples of cross talk at the level of phosphotransfer between histidine kinases and noncognate response regulators (48, 53), cross talk is generally thought not to occur at the level of phosphotransfer between a histidine kinase and its noncognate response regulator, given the selectivity of the interaction between these signaling proteins (54–57) as well as the expected decrease in the specificity of response to an input (54, 58). Here, we present no evidence for cross talk between paralogs but show evidence of cobinding of chemotaxis paralogs that form the signaling baseplate. This organization would allow for the integration and coordination of signaling from two otherwise independent chemotaxis signaling pathways without invoking the potential disadvantages of cross talk.

## MATERIALS AND METHODS

### Strains, media, and chemicals.

The bacterial strains used in this study are listed in Table S2 in the supplemental material. E. coli strains were grown in Luria broth at 37°C supplemented with appropriate antibiotics (concentrations listed in footnotes of Table S2). *A. brasilense* strains were grown on plates at 28°C on minimal medium for *A. brasilense* (MMAB) supplemented with 10 mM malate. Liquid cultures were grown by shaking (200 rpm) at 28°C in MMAB supplemented with 10 mM malate and 18.7 mM ammonium chloride. To induce nitrogen fixation, cells were pelleted and washed three times with MMAB (supplemented with 10 mM malate, no nitrogen) and incubated in 5 ml of MMAB (supplemented with 10 mM malate, no nitrogen) at 28°C without shaking to ensure low aeration for at least 6 h.

10.1128/mBio.01757-19.9 TABLE S2 Primers used in this study. Download Table S2, DOCX file, 0.01 MB. Copyright © 2019 O’Neal et al. 2019 O’Neal et al. This content is distributed under the terms of the Creative Commons Attribution 4.0 International license.

### Construction of *A. brasilense cheA2*::*tet cheA3*::Tn*5* strain.

To generate the *cheA2*::*Tet cheA3*::Tn5 strain, a *cheA2* knockout insertion was produced using a pKNOCK suicide vector carrying an internal fragment of *cheA2* and introduced into the *cheA3*::Tn*5* strain by mating. pKNOCKCheA2 was generated by amplifying an internal fragment of *cheA2* (NCBI accession no. ALJ38472.1) using the primers listed in Table S2. The pKNOCK vector and the PCR fragments were digested with SmaI, ligated, and transformed into competent E. coli TOP10 cells. E. coli TOP10(pKNOCKCheA2) then was used as a donor in conjugation with the *A. brasilense cheA3*::Tn*5* derivative (20). Disruptants were selected on MMAB with tetracycline (10 mg/ml) and confirmed using colony PCR.

### Fluorescence microscopy.

Gateway cloning (Invitrogen) and the pRH005 vector were used to construct all yellow fluorescence protein (YFP) fusions (59). The pRH005 vector allows cloning of any gene to generate products fused in-frame with YFP at their C termini. Most YFP strains used in this study were generated using Gateway technology and adhering to the manufacturer’s protocol (Table S1) (26, 60). Briefly, genes of interest were amplified using specific Gateway primers (Table S2) and *A. brasilense* strain Sp7 genomic DNA. Five microliters of PCR product was run on a 0.8% gel for verification of the insert, and PCR cleanup (Macherey Nagel) was performed on the remainder of the PCR product. Resulting PCR products underwent a BP Clonase (Invitrogen) reaction with the pDONR2.1 vector (Invitrogen). This reaction then was transformed into Top10 chemically competent cells and plated on Luria broth (LB) with kanamycin (50 μg/ml). Colonies from these plates were grown in 5 ml of LB with kanamycin (50 μg/ml), were subjected to plasmid purification (Qiagen), and resulting plasmids underwent an LR reaction (Invitrogen) with the pRH005 plasmid. Resulting reaction mixtures were transformed into Top10 competent cells and plated on LB with kanamycin (50 μg/ml). All constructs were grown in LB with kanamycin, plasmid prepped, and introduced into Sp7 and other strains (Table S2) by biparental mating as described in Hauwaerts et al. (61). One milliliter of cells grown as described above was pelleted at 5,000 rpm for 2 min. Twenty microliters of the pelleted cells was resuspended in MMAB, mounted on a glass slide containing a 100-μl agarose pad (1% low-melting-point agarose in 1× phosphate-buffered saline [PBS] buffer containing 8 g/liter NaCl, 0.2 g/liter KCl, 0.24 g/liter KH_2_PO_4_, 0.144 g/liter Na_2_HPO_4_, pH 7.0), and covered with a cover slip. Cells were left undisturbed for 2 h or, in some instances, overnight before being imaged. Images were captured using a Nikon ECLIPSE 80i fluorescence microscope equipped with a Nikon CoolSnap HQ2 cooled charge-coupled device camera. Measurements were taken from at least 80 cells from three independent cultures, and five fields of view were used for each sample. The results were graphed using GraphPad Prism software and analyzed statistically using two-tailed Z tests to determine if fluorescent focus localization differed significantly from that of the WT in *Δche1*, *Δche4*, and *Δche1 Δche4* strains.

10.1128/mBio.01757-19.8 TABLE S1 Strains used in this study. Download Table S1, DOCX file, 0.02 MB. Copyright © 2019 O’Neal et al. 2019 O’Neal et al. This content is distributed under the terms of the Creative Commons Attribution 4.0 International license.

### Bacterial two-hybrid assay.

A bacterial two-hybrid assay specific for membrane proteins was used to investigate protein-protein interactions (76). In this assay, genes of interest were cloned into either pKNT25 (low-copy-number) or pUT18 (high-copy-number) vector. These vectors each encode one-half of a catalytic domain (T18 or T25) of the Bordetella pertussis adenylate cyclase. If protein-protein interaction takes place, functional complementation occurs between the two halves of the catalytic domain and cyclic AMP (cAMP) is produced. This activates the *lac* and *mal* operons in E. coli; positive interactions plated on MacConkey agarose utilize the carbon source in the agar and appear pink. From here, pink colonies can be grown in liquid medium and subjected to a beta-galactosidase assay to quantify the strength of interactions. Proteins of interest (CheA1, CheA4, CheA2, CheA3, CheW1, CheW4, Tlp1, and Tlp4a) were fused on the C terminus of the T18 and T25 domains of Bordetella pertussis adenylate cyclase present in vectors pUT18 and pKNT25, respectively, as described by the manufacturer’s protocol (Euromedex). The genes of interest were first PCR amplified (Table S3) and cloned into a TOPO 2.1 vector (Invitrogen). The resulting vectors were digested with enzyme pairs (HindIII and EcoRI for *cheA4*, *cheW1*, *cheW4*, *tlp1*, and *tlp4a*; HindIII and KpnI for *cheA1*) and ligated into their destination vectors (high-copy-number pUT18 and low-copy-number pKNT25) (Table S1) that were previously digested with the same enzymes using T4 ligation (New England Biolabs). *cheA2* and *cheA3* were introduced into the Gateway-compatible versions of the pUT18 and pKNT25 vectors (61), using primers listed in Table S2 and manufacturer’s protocols. Resulting plasmids were propagated in XL-1 Blue cells (Agilent Technologies), and the presence of an insert was confirmed by colony PCR. To test for protein-protein interactions, two plasmids expressing genes of interest were cotransformed into BTH101 competent cells and plated on LB plates with kanamycin (50 μg/ml) and carbenicillin (50 μg/ml). The plates were incubated for 48 h at 30°C. Several colonies were picked from a plate, inoculated into 5 ml of liquid LB with kanamycin and carbenicillin (50 μg/ml of each), and shaken (200 rpm) at 30°C. Two microliters of the overnight cultures were spotted onto MacConkey plates with lactose as a carbon source and incubated at 30°C for up to 96 h. Empty vectors (pUT18 and pKNT25) were used as negative controls, while pUT18-zip and pKT25-zip (62) were used as positive controls. For quantification of interactions, cells were grown in 5 ml liquid LB with kanamycin and carbenicillin (50 μg/ml of each) at 30°C with shaking at 200 rpm until they reached an optical density at 600 nm (OD_600_) of 0.5 to 0.6. A beta-galactosidase assay then was performed as described in Ramsay et al. (63).

### Protein pulldown.

Protein pulldowns were used to confirm protein-protein interactions identified in the bacterial 2-hybrid assay. Gateway cloning and pDEST24 (Invitrogen) were used to generate CheA1ΔTMX-GST and CheA4-GST. Two liters of BL21(DE3) (pDEST CheA1ΔTMX-GST) and BL21(DE3) (pDEST CheA4-GST) cells were grown to an OD_600_ of 0.5 and induced with 1 mM isopropyl-β-d-thiogalactopyranoside (IPTG) for 3 h. Cells were collected and washed with 1× PBS (pH 8.0). Pellets were resuspending in radioimmunoprecipitation assay (RIPA) buffer (50 mM Tris-HCl, 150 mM NaCl, 1% Triton X-100, 0.1% SDS, 1 mM phenylmethylsulfonyl fluoride, pH 8.0) and lysed using a French press. Cell debris was removed by centrifuging at 17,000 rpm for 1 h at 4°C. Total protein concentration was quantified using a Bradford device. Lysate (2 mg total protein) was then applied to an equilibrated 2-ml glutathione agarose resin and incubated together for 4 h with rotation. Unbound proteins were washed off with 5 bed volumes of PBS. YFP-tagged CheA1ΔTMX-YFP, CheA4-YFP, Tlp1-YFP, Tlp4a-YFP, CheW1-YFP, or CheW4-YFP was expressed in the corresponding mutant backgrounds, and 500 ml was grown to an OD_600_ of 0.8. Protein expression was confirmed using fluorescence microscopy. Cells were collected via centrifugation (6,000 rpm for 20 min at 4°C) and lysed in RIPA buffer using sonication with lysozyme (10 cycles of 15-s bursts followed by 10-s rest). Protein concentration was quantified using a Bradford assay (Bio-Rad). Whole-cell lysate (2 mg total protein) was applied to the previously prepared CheA1ΔTMX/CheA4-GST resin and incubated overnight at 4°C with rotation. Unbound proteins were eluted using 10 bed volumes of 1× PBS. Column-bound proteins were eluted with 1× PBS containing 10 mM glutathione. Protein interactions were confirmed using Western blotting as previously described, with the following exception: YFP-tagged proteins were detected using anti-green fluorescent protein (anti-GFP) antibody (Abcam) at a 1:1,000 dilution. CheA1ΔTMX-GST and CheA4-GST column binding was confirmed using anti-glutathione *S*-transferase (anti-GST) polyclonal antibody (1:1,000) (Invitrogen). The membranes were incubated with a 1:5,000 dilution of IRDye CW 800 donkey anti-rabbit and developed using an Odyssey device (LiCor).

### Electron cryotomography.

*Azospirillum* cultures were grown overnight in 5 ml MMAB + N (with ammonium chloride) with 200 μg/ml ampicillin at room temperature without shaking. The culture was spun down for 10 min at 3,500 × *g*, and the pellet was resuspended in 5 ml MMAB – N (without ammonium chloride) and spun down again for 10 min at 3,500 × *g*. The pellet then was resuspended in 5 ml MMAB − N with 200 μg/ml ampicillin and left at room temperature (on bench) without shaking overnight. Cells were prepared on EM grids as described previously (17). Images were collected on either an G2 300-keV field emission gun microscope or TITAN Krios microscope with lens aberration correction (both from FEI, now part of Thermo Fischer Scientific). Both microscopes were equipped with GATAN K2 summit-counting electron detector cameras and GATAN imaging filters. Data were collected using the UCSFtomo software, using a cumulative dose of ∼160 e/Å^2^. Tomograms were constructed automatically using either RAPTOR (64), a program embedded in the Jensen laboratory pipeline and database (65), or the imod software package (66).

### Measuring the height of chemoreceptor arrays.

To measure the distance between the inner membrane and the CheA/CheW base plate, we used 3dmod, v4.9.9 (66), to mark the inner membrane above the chemoreceptor array with model points. We next used a custom script written in Node.js that takes as input the tomogram and the model points to calculate the average pixel value in profiles running perpendicular to the model points but in the same plane as the model points to collect and generate a JSON-formatted file with the average pixel intensity of each profile. Each file generated was named after the name of the tomogram, the name of the model, and a unique number in case there was more than one profile per tomogram. The script is available in the GitLab repository at https://gitlab.com/daviortega/sideview-profile-average.

To visualize the profiles, we used the ObservableHQ notebook located at https://observablehq.com/@daviortega/generic-notebook-to-analyse-1d-averaged-electron-density-p. For each profile, we measured the distance between the dips corresponding to the electron density of the inner membrane and the CheA/CheW baseplate in pixels. Uncertainty was estimated based on the precision to determine the center of each dip in pixels and propagated to the measure of distance between dips. We summarized these results and additional information in Table S3 and Fig. S3, S4, and S5 to show the distribution of height in the analyzed tomograms. Each measurement is reported with the value and their respective uncertainty, with coverage (K) of 2, because the number of degrees of freedom in the measurement is large.

### Bioinformatics data sets and analysis.

*A. brasilense* paralogs were aligned using BLAST Global align to identify sequence similarity. Residues 446 to 506 of CheA1 (accession no. AAL47021.1) and residues 334 to 397 of CheA4 (accession no. WP_059399028.1) were used for aligning P3 domains. For P5 domains, residues 698 to 834 (P5A) and 860 to 984 (P5B) of CheA1 and residues 543 to 675 of CheA4 were aligned. The entire sequences of CheW1 (AAL47022.1) and CheW4 (WP_035675900.1) were aligned. To identify conserved residues, the BLAST multiple alignment tool was used to align CheA1 P5A and P5B domains with residues 519 to 659 of E. coli CheA (accession no ANK01864.1). For CheW alignments, full-length CheW1 and CheW4 of *A. brasilense* were aligned with E. coli CheW (accession no. ANK01863.1). Residues necessary for CheA-CheW interactions were identified based on references 35 and 37.

To predict the assignment of chemoreceptors to polar arrays, the chemoreceptor sequences were first collected from MiST (67). Multiple-sequence alignments were conducted using the L-INS-I algorithm from the MAFFT package, version v7.305b (68), or T-COFFEE, version_11.00.d625267 (69), and manually edited using Jalview, v2.10.1 (70). The alignments were manually edited only at the region between the transmembrane and the signaling domain. In the multiple-sequence alignments, we used locus number as sequence headers. Secondary structure prediction was conducted using Jpred4 (71) To build homology models we used MODELLER, v9.21 (72). Domain architecture of chemoreceptors was produced using CDVist (73). Measurements performed in atomic models from the PDB (74) were obtained using VMD v1.9.1 (75).

### Assignment of chemoreceptors encoded by the *A. brasilense* genome to arrays of specific height.

The height of chemoreceptor arrays is determined by the physical length of membrane-bound chemoreceptor proteins present in the array, which in turn can be predicted from sequence based on the heptad class of the signaling domain (18) and the arrangement of protein domains present between the transmembrane region and the signaling domain (11). Twenty-eight-nanometer chemotaxis arrays have been reported to belong to 38H receptors with extra alpha helix linkers in *M. magneticum* (11). The multiple-sequence alignment of sequences of 38H chemoreceptors from both *A. brasilense* and *M. magneticum* does not contain major gaps in the region between the transmembrane and the signaling domain, which suggests that they share the same domain architecture (Data set S1 at doi:10.6084/m9.figshare.9714056). As in a few chemoreceptors in *M. magneticum*, two 38H receptors in *A. brasilense* have an extra HAMP instead of the alpha helix linker (AMK58_RS04445 and AMK58_RS08090). Based on the alignment, the alpha helix linker has approximately the same number of residues as one of the HAMP helices (Fig. S3). These results suggest that the short arrays visualized in the wild-type cells are formed by 38H chemoreceptors with either domain structure. The *A. brasilense* genome contains three 36H chemoreceptors, and all of them have 3 HAMP domains. In addition, the chemoreceptor present in the *che4* operon, *tlp4a*, also has a PAS domain between the first and second HAMP domains counting from the transmembrane. To predict the height of chemoreceptors, we built 100 homology models using MEALZ_2872 from Methylomicrobium alcaliphilum as a template (32) with MODELLER. MODELLER was configured to have library_schedule = autoschedule.slow, max_var_interations = 1000, md_leve = refine.slow, repeat optimization = 100, and max_molpdf = 1e6. The alignment was built using T-Coffee. Based on the secondary structure predictions we restrained parts of the sequence to be alpha helical from residues 302 to 343 and 258 to 285, and we added restraints with respect to stereochemical constraints.
