# Supplementary material for: Distinct Chemotaxis Protein Paralogs Assemble into Chemoreceptor Signaling Arrays To Coordinate Signaling Output
Source: mBio. 2019 Sep 24;10(5):e01757-19. doi: 10.1128/mBio.01757-19 (PMC6759762; doi:10.1128/mBio.01757-19)
Supplement: FIG S2 [file mBio.01757-19-sf002.pdf]

A.

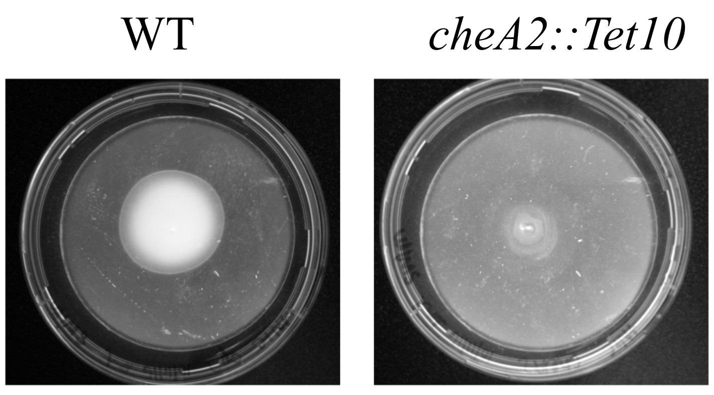

B.

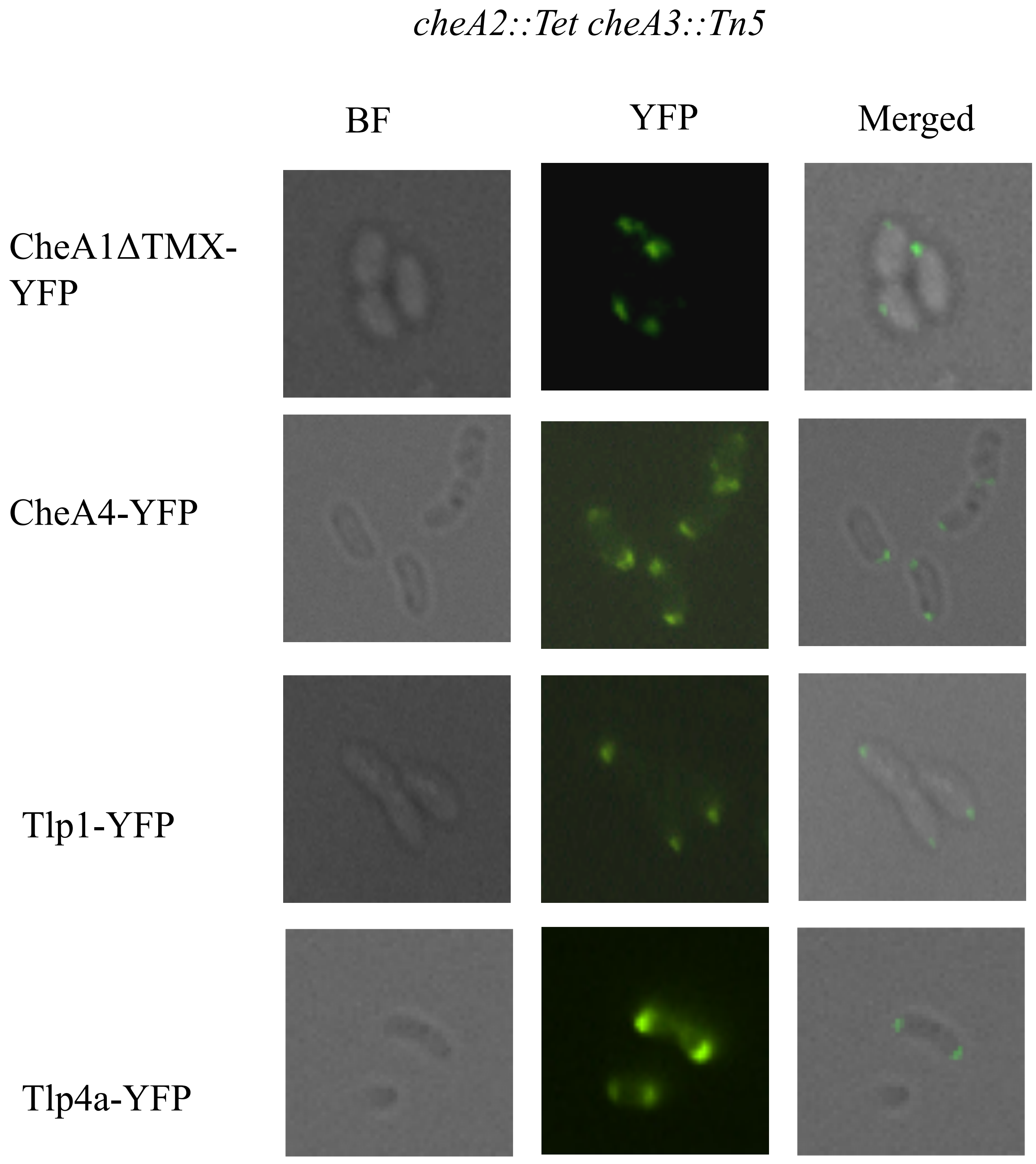

C.

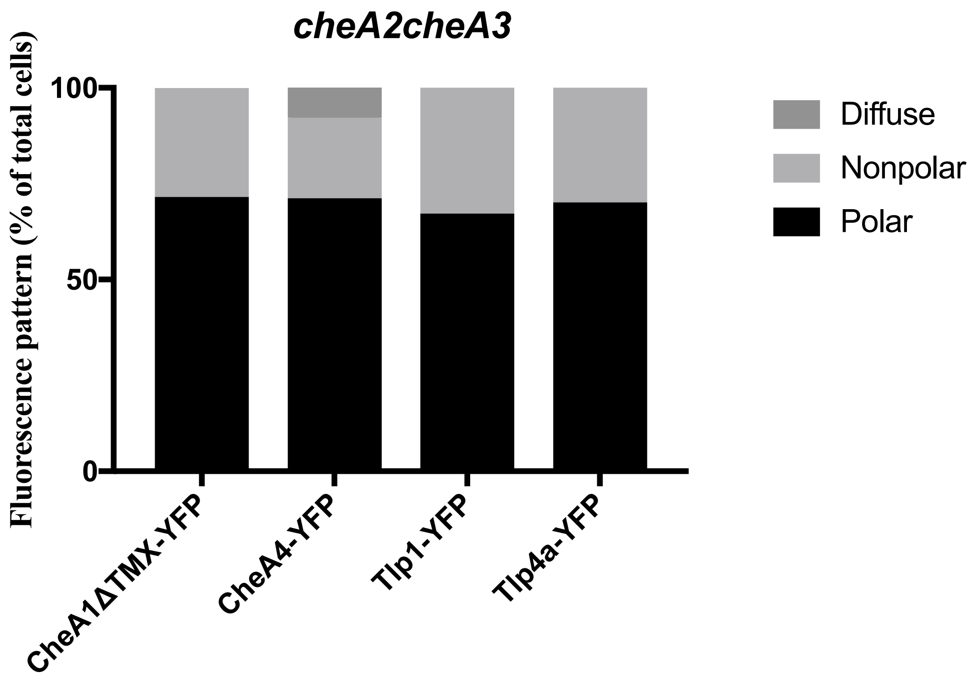

D.

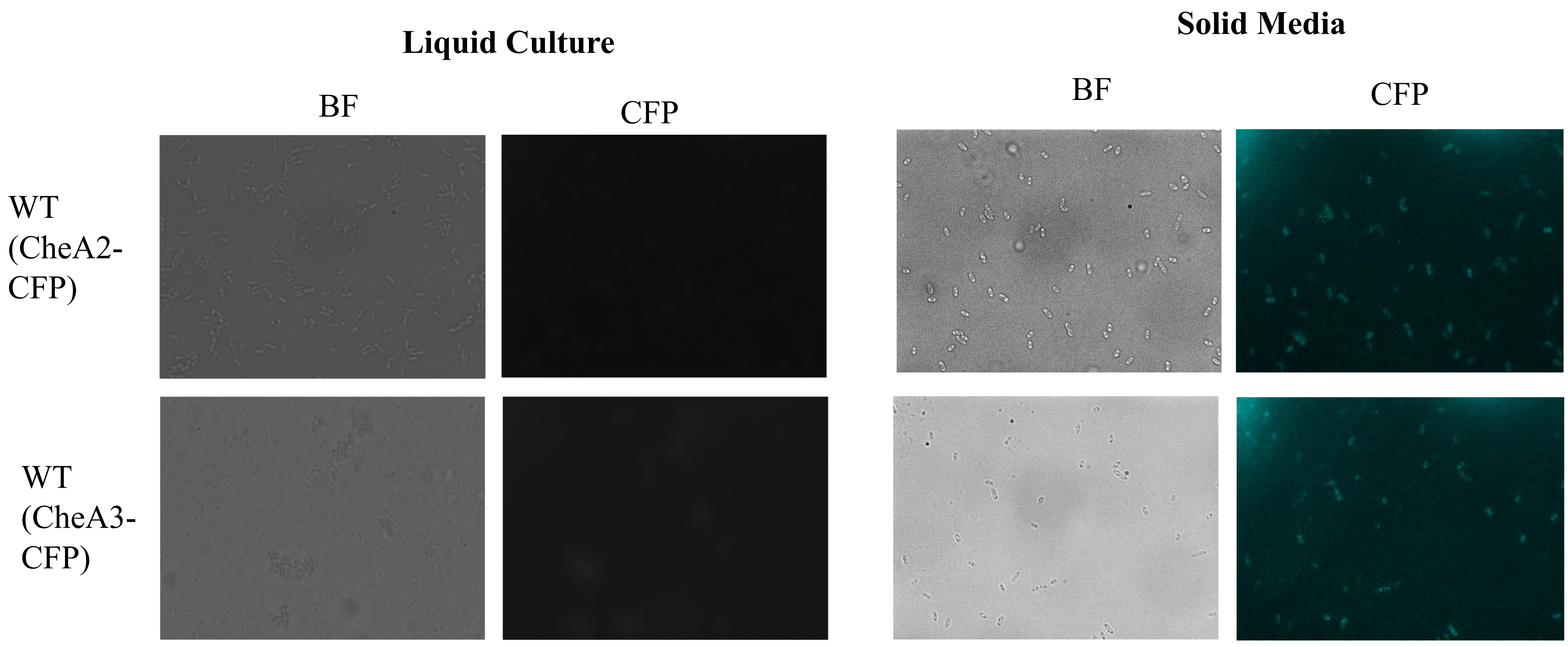

Fig S2: Involvement of CheA2 and CheA3 in chemotaxis and array formation.Cells lacking CheA2 are impaired in swimming in a soft agar assay (A). Localization of chemotaxis proteins in *cheA2:: Tet cheA3::Tn5* background. YFP tagged CheA1ΔTMX, CheA4, Tlp1, and Tlp4a are all able to polarly localize in the absence of CheA2 and CheA3 (B-C). Localization of CheA2-CFP and CheA3-CFP when grown in liquid or on solid media.
