# Supplementary figures and images for: Distinct Chemotaxis Protein Paralogs Assemble into Chemoreceptor Signaling Arrays To Coordinate Signaling Output
Source: mBio. 2019 Sep 24;10(5):e01757-19. doi: 10.1128/mBio.01757-19 (PMC6759762; doi:10.1128/mBio.01757-19)

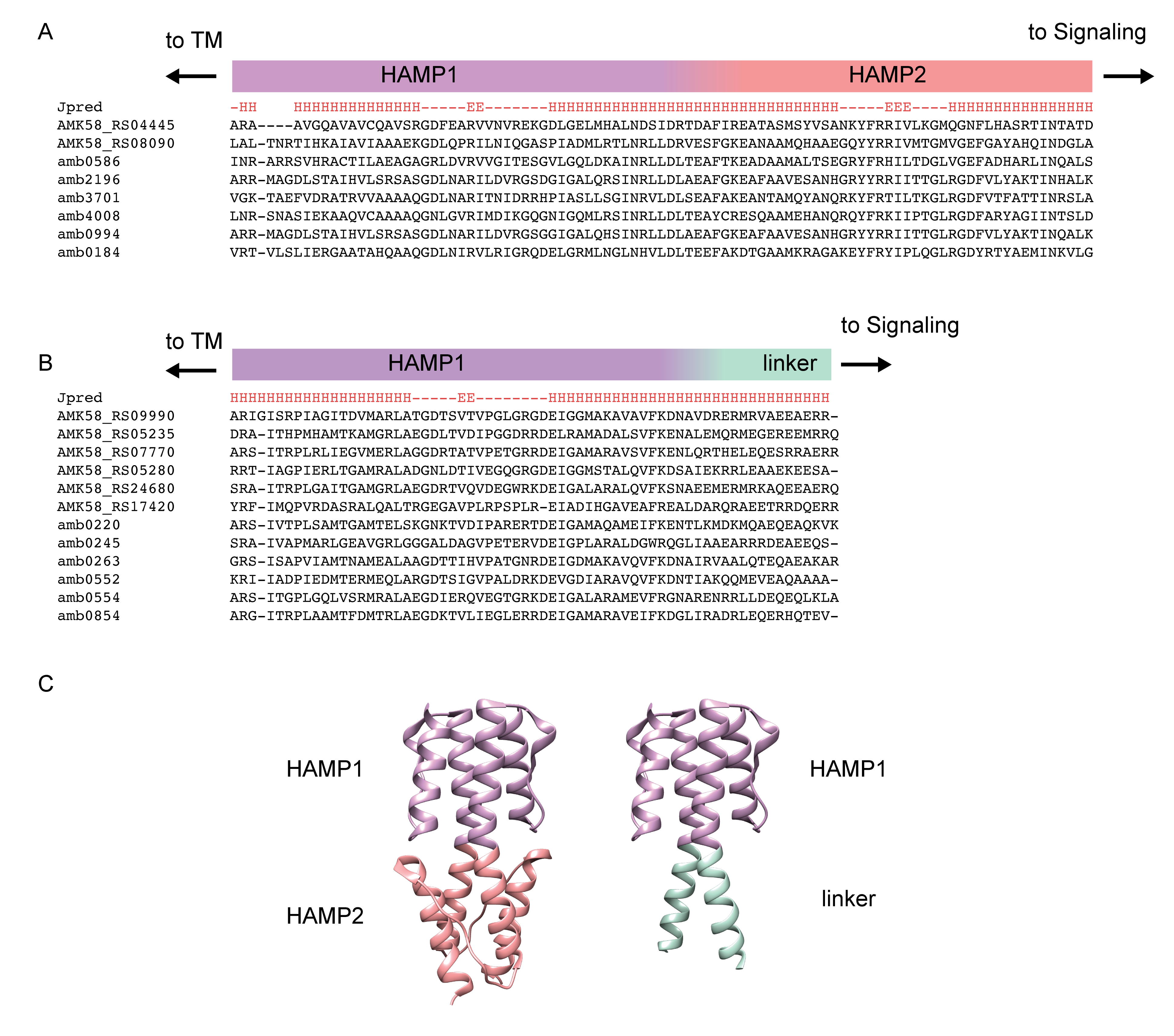

Supplement: FIG S3 [file mBio.01757-19-sf003.tif]

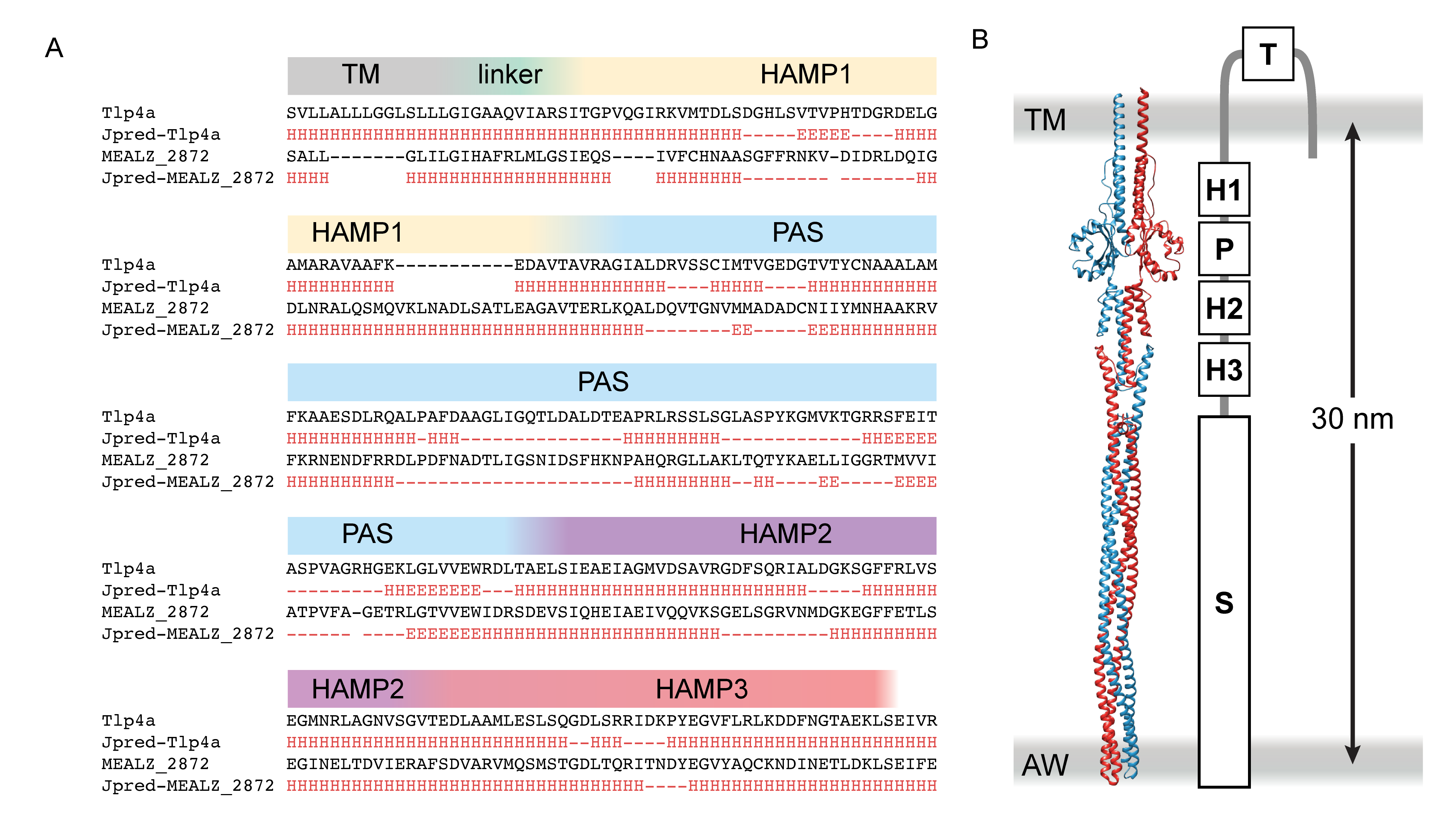

Supplement: FIG S4 [file mBio.01757-19-sf004.tif]

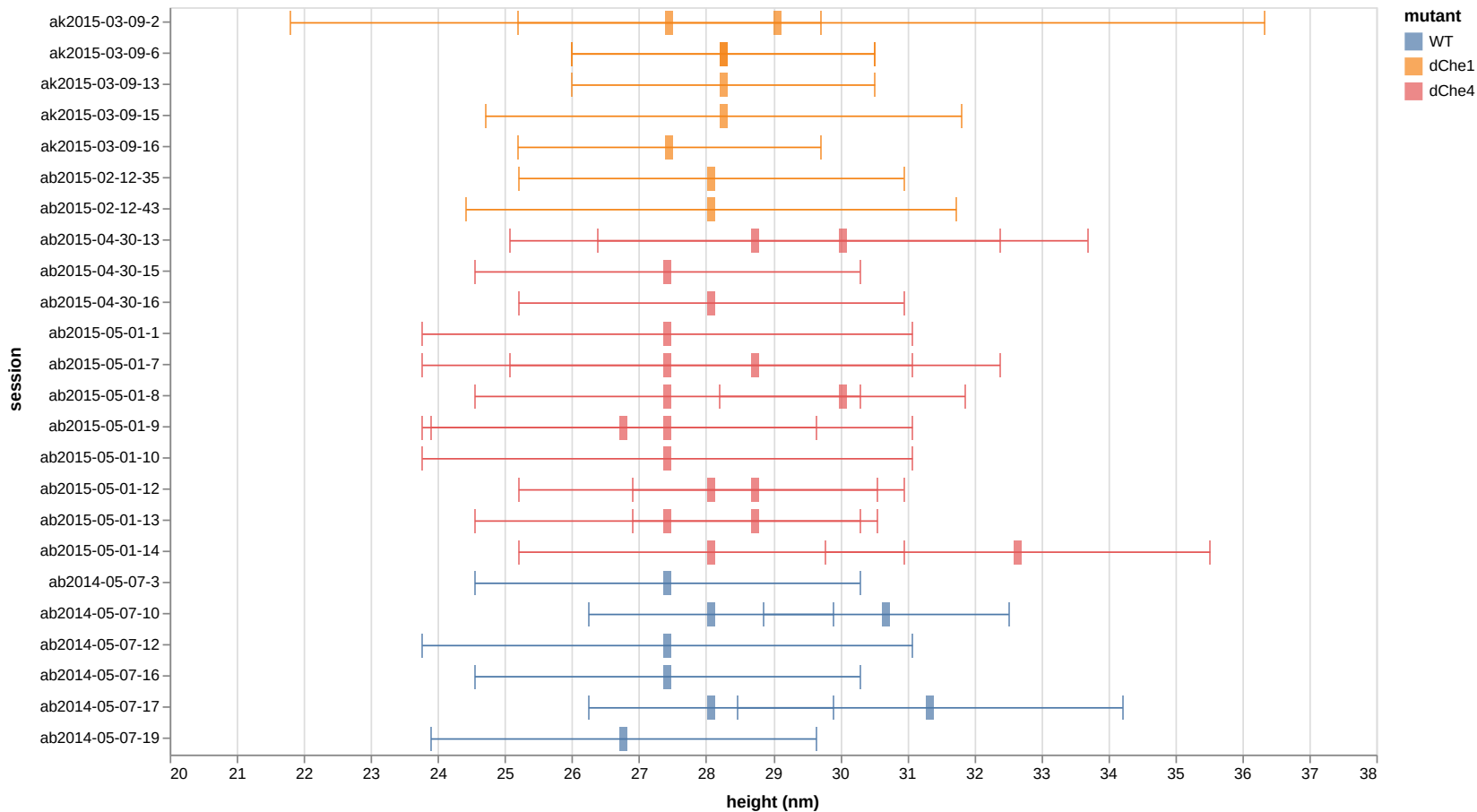

Supplement: FIG S5 [file mBio.01757-19-sf005.pdf]
