# Supplementary material for: Distinct Chemotaxis Protein Paralogs Assemble into Chemoreceptor Signaling Arrays To Coordinate Signaling Output
Source: mBio. 2019 Sep 24;10(5):e01757-19. doi: 10.1128/mBio.01757-19 (PMC6759762; doi:10.1128/mBio.01757-19)
Supplement: TABLE S3 [file mBio.01757-19-st003.docx]

**Table S3: Summary of measured height of chemoreceptor arrays.**

| **mutant** | **session** | **tomogram** | **pixel size (nm)** | **model ID** | **height (pixel)** | **uncertainty (pixel)** |
| --- | --- | --- | --- | --- | --- | --- |
| Δ*che1* | ak2015-03-09-2 | atlas10002_full.rec | 1.615 | 1 | 17 | 1.4 |
| Δ*che1* | ak2015-03-09-2 | atlas10002_full.rec | 1.615 | 2 | 18 | 4.5 |
| Δ*che1* | ak2015-03-09-6 | atlas10006_full.rec | 1.615 | 3 | 17.5 | 1.4 |
| Δ*che1* | ak2015-03-09-6 | atlas10006_full.rec | 1.615 | 4 | 17.5 | 1.4 |
| Δ*che1* | ak2015-03-09-13 | atlas10013_part121_4.rec | 1.615 | 5 | 17.5 | 1.4 |
| Δ*che1* | ak2015-03-09-15 | atlas10015_part121_3.rec | 1.615 | 6 | 17.5 | 2.2 |
| Δ*che1* | ak2015-03-09-16 | atlas10016_full.rec | 1.615 | 7 | 17.0 | 1.4 |
| Δ*che1* | ab2015-02-12-35 | AzoDeltaOp1Feb0006.rec | 1.306 | 8 | 21.5 | 2.2 |
| Δ*che1* | ab2015-02-12-43 | AzoDeltaOP1secondset0001_full.rec | 1.306 | 9 | 21.5 | 2.8 |
| Δ*che4* | ab2015-04-30-13 | deltaOp4april0001_full.rec | 1.306 | 10 | 23 | 2.8 |
| Δ*che4* | ab2015-04-30-13 | deltaOp4april0001_full.rec | 1.306 | 11 | 22 | 2.8 |
| Δ*che4* | ab2015-04-30-15 | deltaOp4april0003_full.rec | 1.306 | 12 | 21 | 2.2 |
| Δ*che4* | ab2015-04-30-16 | deltaOp4april0004_full.rec | 1.306 | 13 | 21.5 | 2.2 |
| Δ*che4* | ab2015-05-01-1 | deltaOp4april0020_part121_10.rec | 1.306 | 14 | 21 | 2.8 |
| Δ*che4* | ab2015-05-01-7 | DeltaOp4Day2May0002_full.rec | 1.306 | 15 | 21 | 2.8 |
| Δ*che4* | ab2015-05-01-7 | DeltaOp4Day2May0002_full.rec | 1.306 | 16 | 22 | 2.8 |
| Δ*che4* | ab2015-05-01-8 | DeltaOp4Day2May0003_full.rec | 1.306 | 17 | 21 | 2.2 |
| Δ*che4* | ab2015-05-01-8 | DeltaOp4Day2May0003_full.rec | 1.306 | 18 | 23 | 1.4 |
| Δ*che4* | ab2015-05-01-9 | DeltaOp4Day2May0004_full.rec | 1.306 | 19 | 20.5 | 2.2 |
| Δ*che4* | ab2015-05-01-9 | DeltaOp4Day2May0004_full.rec | 1.306 | 20 | 21 | 2.8 |
| Δ*che4* | ab2015-05-01-10 | DeltaOp4Day2May0005_part121_34.rec | 1.306 | 21 | 21 | 2.8 |
| Δ*che4* | ab2015-05-01-12 | DeltaOp4Day2May0007_part121_25.rec | 1.306 | 22 | 21.5 | 2.2 |
| Δ*che4* | ab2015-05-01-13 | DeltaOp4Day2May0008_part121_10.rec | 1.306 | 23 | 21 | 2.2 |
| Δ*che4* | ab2015-05-01-13 | DeltaOp4Day2May0008_part121_10.rec | 1.306 | 24 | 22 | 1.4 |
| Δ*che4* | ab2015-05-01-14 | DeltaOp4Day2May0010_part121_19.rec | 1.306 | 25 | 21 | 2.2 |
| Wild type | ab2014-05-07-3 | AzoWild type0003_full.rec | 1.306 | 27 | 21 | 2.2 |
| Wild type | ab2014-05-07-10 | AzoatlasBnew0001_full.rec | 1.306 | 28 | 23.5 | 1.4 |
| Wild type | ab2014-05-07-10 | AzoatlasBnew0001_full.rec | 1.306 | 29 | 21.5 | 1.4 |
| Wild type | ab2014-05-07-12 | AzoatlasBnew0003_full.rec | 1.306 | 30 | 21 | 2.8 |
| Wild type | ab2014-05-07-16 | AzoatlasBnew0007_full.rec | 1.306 | 31 | 21 | 2.2 |
| Wild type | ab2014-05-07-17 | AzoatlasBnew0008_full.rec | 1.306 | 32 | 21.5 | 1.4 |
| Wild type | ab2014-05-07-17 | AzoatlasBnew0008_full.rec | 1.306 | 33 | 24 | 2.2 |
| Wild type | ab2014-05-07-19 | AzoAtlasCMay0003_full.rec | 1.306 | 34 | 20.5 | 2.2 |
