# Supplementary material for: Distinct Chemotaxis Protein Paralogs Assemble into Chemoreceptor Signaling Arrays To Coordinate Signaling Output
Source: mBio. 2019 Sep 24;10(5):e01757-19. doi: 10.1128/mBio.01757-19 (PMC6759762; doi:10.1128/mBio.01757-19)
Supplement: FIG S7 [file mBio.01757-19-sf007.docx]

**A.**

AbCheA1P5A --------------------------------------------SALIVE-CAGERFAIP 15
AbCheA1P5B --------------------------------------------ALLLFRAGAGAPKGVP 16
AbCheA4P5 SGRGVGMDVVRRNISSLGGRIGVYSTPGEGSRFVLSLPLTLAVLDGMVIS-VGEERFVLP 59
 E.coliP5 --------------------------------IRILLPLTLAILDGMSVR-VADEVFILP 27
 : . . :*

AbCheA1P5A QISVVELVRAAADSEHTIERLKGTPVLRLRNRLLPLVSLQELLRLDDQEGGEKKTDETFI 75
AbCheA1P5B LSLV---ARLEDVDLASVESSNGMPVVQYRGKLMPLVPIDPGFMIGQEG-----R--QPV 66
AbCheA4P5 LTNIVESLRPKAADLHGL--VGKCDVMMARGEYVRLVYLHQLFGIPGAVA--DPTRALVV 115
 E.coliP5 LNAVMESLQPREADLHPL--AGGERVLEVRGEYLPIVELWKVFNVAGAKT--EATQGIVV 83
 : : . : *: *.. : :* : : : :

AbCheA1P5A VVTQVGTYTFGIMVDRVFDTEEIVVKPVAPILRHIEMFSGNTIFGDGSVIMILDPNGIAS 135
AbCheA1P5B LVFADGDRSMGLIVDEIVDIVEERL--VEQLTADRPGLMGSAIIAGKAT-DVLDAGFFLT 123
AbCheA4P5 LVETEDGSRLGLVVDEVL------------------------------------------ 133
 E.coliP5 ILQ-SGGRRYALLVDQLIGQHQVVVKNLESNYRKVPGISAATILGDGSVALIVDVSALQ- 141
 :: . .::**.:.

AbCheA1P5A A 136
AbCheA1P5B Q 124
AbCheA4P5 -
 E.coliP5 -

**B.**

AbCheW1 MSNAKLPATT----KKSKG-DEITSGGSQDYVTMTIADQLFGIPVLQVQDVLGHQRITRI 55
AbCheW4 MSSSTALATVGGSRTDARNDVMAVNAAEEQYVTFTVGSEEYGVNILSVREIRGWTPESRL 60
EcoliCheW ---------MTG---MTNVTKLASEPSGQEFLVFTLGDEEYGIDILKVQEIRGYDQVTRI 48
 :. . . ::::.:*:..: :*: :*.*::: * :*:

AbCheW1 PLAPPEVAGSLNLRGRIVTAIDVRLRLGLTSRPKDKPGMSIVVDLRGELYSLMVDSVGEV 115
AbCheW4 PNLPDYVRGVINLRGIIIPIFDLRARFGGGATSVTKRHVVVVIQVGERTRGILVDAISDI 120
EcoliCheW ANTPAFIKGVTNLRGVIVPIVDLRIKFSQVDVDYNDNTVVIVLNLGQRVVGIVVDGVSDV 108
 * : * **** *: .*:* ::. . : :*::: . .::**.:.::

AbCheW1 LSLSNDDFERNPATLD-PRWREVSTGIYRLNGQLMVVLDVPRLLNFTTMEAA-------- 166
AbCheW4 LAIGHDAIKPPPDVDGGMVDAEYLSGLYTADDRMVTLLCVEKLFSVENSELD-SATKALP 179
EcoliCheW LSLTAEQIRPAPEFAV-TLSTEYLTGLGALGDRMLILVNIEKLLNSEEMALLDSAASEVA 167
 *:: : :. * * :*: ..::: :: : :*:.

AbCheW1 ------
AbCheW4 ALERAS 185
EcoliCheW ------

**Fig. S7.** Sequence alignment between *A. brasilense* chemotaxis paralogs and *E. coli* homologs. A) P5 domains of *A. brasilense* CheA1, CheA4, and E. coli CheA. Highlighted residues are conserved residues necessary for interaction with CheW in *E. coli*. B) Alignment of *A. brasilense* CheW1 and CheW4 with *E. col*i CheW. Highlighted residues are essential for CheA-CheW interaction in *E. coli*. “* “ indicates residue at this position is fully conserved, “: “ indicates residues are highly similar, and “.” indicates residues are weakly similar.
