# Supplementary material for: Distinct Chemotaxis Protein Paralogs Assemble into Chemoreceptor Signaling Arrays To Coordinate Signaling Output
Source: mBio. 2019 Sep 24;10(5):e01757-19. doi: 10.1128/mBio.01757-19 (PMC6759762; doi:10.1128/mBio.01757-19)
Supplement: TABLE S2 [file mBio.01757-19-st002.docx]

**Table S2. Primers used in this study.**

| **Primer name** | **Sequence** |
| --- | --- |
| CheA4 HindIII For BACTH | 5’ - AAG CTT ATG GAC GGG GTG CGC AAC AC - 3’ |
| CheA4 EcoRI Rev BACTH | 5’ - GAA TTC GAC CGG TTC GAG TGC GGG GGC - 3’ |
| Tlp4a HindIII For BACTH | 5’ - AAG CTT ATG GCG AAA GGG GTC GGT TCG - 3’ |
| Tlp4a EcoRI Rev BACTH | 5’ - GAA TTC TGC CGC CCG TCC GCG GGC CAG - 3’ |
| Tlp4S HindIII Fwd BACTH | 5’ -AAG CTT ATG CTC GCC CAC GAG GGC GC-3’ |
