# Supplementary material for: Distinct Chemotaxis Protein Paralogs Assemble into Chemoreceptor Signaling Arrays To Coordinate Signaling Output
Source: mBio. 2019 Sep 24;10(5):e01757-19. doi: 10.1128/mBio.01757-19 (PMC6759762; doi:10.1128/mBio.01757-19)
Supplement: TABLE S1 [file mBio.01757-19-st001.docx]

**Table S1. Strains used in this study.**

| **Strains or Plasmids** | **Genotype, relevant characteristics** | **Reference/source** |
| --- | --- | --- |
| *Strains* |  |  |
| *E. coli* Top10 | General cloning strain | Invitrogen |
| *E. coli* BTH101 | F^−^ *cya-99 araD139 galE15 galK16 rpsL1 hsdR2 mcrA1 mcrB1* | (58) |
| *E. coli*  XL-1 Blue | *recA1 endA1 gyrA96 thi-1 hsdR17 supE44 relA1 lac* [F´ *proAB lacIq*  *Z∆M15* Tn*10* (Tet^r^ )]. | Agilent Technologies |
| *E. coli*  S17.1 | *thi endA recA hsdR* with RP4-2Tc::Mu-Km::Tn*7* integrated in chromosome | (63) |
| *A. brasilense* Sp7 | Wild type *A. brasilense* | ATCC29145 |
| *A. brasilense* *∆che1* | *∆(cheA1-cheR1):: Cm (Cm^r^)* | (23) |
| *A. brasilense* *∆che4* | *∆(cheA4-cheR4):: Gm (Gm^r^)* | (21) |
| *A. brasilense* *∆che1∆che4* | *∆(che1)∆(che4):: Cm (Cm^r^), Gm (Gm^r^)* | (21) |
| *A. brasilense* *∆cheA1∆cheA4* | *∆(cheA1)∆(chAe4):: gusA-Km (Km^r^), Gm (Gm^r^)* | (21) |
| *A. brasilense* *∆cheA1* | *∆(cheA1):: gusA-Km (Km^r^)* | (23) |
| *A. brasilense* *∆cheA4* | *∆(cheA4):: Gm (Gm^r^)* | (21) |
| *A. brasilense* *cheA2::Tet cheA3::Tn5* | *cheA2::Tet (Tet^r^) cheA3:: Tn5 (Km^R^)* | This work |
| *A. brasilense* *∆che1* (prhCheA1∆TMX) | Sp7 mutant derivative of *A. brasilense* deleted for *che1* with a plasmid containing CheA1∆TMX | This work |
| *A. brasilense*  *∆che4* (prhCheA1∆TMX) | Sp7 mutant derivative of *A. brasilense* deleted for *che4* with a plasmid containing CheA1∆TMX | This work |
| *A. brasilense*  *∆che1∆che4* (prhCheA1∆TMX) | Sp7 mutant derivative of *A. brasilense* deleted for *che1 and che4* with a plasmid containing CheA1∆TMX | This work |
| *A. brasilense*  *∆che1* (prhCheA4) | Sp7 mutant derivative of *A. brasilense* deleted for *che1* with a plasmid containing CheA4 | This work |
| *A. brasilense*  *∆che4* (prhCheA4) | Sp7 mutant derivative of *A. brasilense* deleted for *che4* with a plasmid containing CheA4 | This work |
| *A. brasilense*  *∆che1∆che4* (prhCheA4) | Sp7 mutant derivative of *A. brasilense* deleted for *che1 and che4* with a plasmid containing CheA4 | This work |
| *A. brasilense*  *∆cheA1* (prhTlp1) | Sp7 mutant derivative of *A. brasilense* deleted for *cheA1* with a plasmid containing Tlp1 | This work |
| *A. brasilense*  *∆cheA4* (prhTlp1) | Sp7 mutant derivative of *A. brasilense* deleted for *cheA4* with a plasmid containing Tlp1 | This work |
| *A. brasilense*  *∆cheA1∆cheA4* (prhTlp1) | Sp7 mutant derivative of *A. brasilense* deleted for *cheA1 and cheA4* with a plasmid containing Tlp1 | This work |
| *A. brasilense*  *∆cheA1* (prhTlp4a) | Sp7 mutant derivative of *A. brasilense* deleted for *cheA1* with a plasmid containing Tlp4a | This work |
| *A. brasilense*  *∆cheA4* (prhTlp4a) | Sp7 mutant derivative of *A. brasilense* deleted for *cheA4* with a plasmid containing Tlp4a | This work |
| *A. brasilense*  *∆cheA1∆cheA4* (prhTlp4a) | Sp7 mutant derivative of *A. brasilense* deleted for *cheA1 and cheA4* with a plasmid containing Tlp4a | This work |
| *A. brasilense*  Sp7 (prhTlp1) | Wild type *A. brasilense* with a plasmid containing Tlp1 | This work |
| *A. brasilense*  Sp7 (prhTlp4a) | Wild type *A. brasilense* with a plasmid containing Tlp1 | This work |
| *A. brasilense*  *∆tlp1* (prhTlp4a) | Sp7 mutant derivative of *A. brasilense* deleted for *tlp1* and containing a plasmid with Tlp4a | This work |
| *A. brasilense*  *∆cheA1* (prh005) | Sp7 mutant derivative of *A. brasilense* deleted for *cheA1* and containing an empty pRH005 plasmid | This work |
| *A. brasilense*  *∆tlp1* (prhTlp1) | Sp7 mutant derivative of *A. brasilense* deleted for *tlp1* and expressing a plasmid containing Tlp1 | (25) |
| *A. brasilense* Sp7 (pKNOCKCheA2) | Wild type A. brasilense with a plasmid containing CheA2 for insertional mutagenesis | This work |
| *A. brasilense*  *cheA2::TetR cheA3::Tn5* (prhCheA1TMX) | Sp7 mutant derivative of *A. brasilense* with Tet insertion into cheA2 gene and Tn5 insertion into cheA3 gene and carrying a plasmid containing CheA1∆TMX | This work |
| *A. brasilense*  *cheA2::TetR cheA3::Tn5* (prhCheA4) | Sp7 mutant derivative of *A. brasilense* with Tet insertion into cheA2 gene and Tn5 insertion into cheA3 gene and carrying a plasmid containing CheA4 | This work |
| *A. brasilense*  *cheA2::TetR cheA3::Tn5* (prhTlp1) | Sp7 mutant derivative of *A. brasilense* with Tet insertion into cheA2 gene and Tn5 insertion into cheA3 gene and carrying a plasmid containing Tlp1 | This work |
| *A. brasilense*  *cheA2::TetR cheA3::Tn5* (prhTlp4a) | Sp7 mutant derivative of *A. brasilense* with Tet insertion into cheA2 gene and Tn5 insertion into cheA3 gene and carrying a plasmid containing Tlp4a | This work |
| *Plasmids* |  |  |
| TOPO 2.1 | PCR cloning vector, Km | Invitrogen |
| pRH005 | Gateway-based destination vector expressing proteins fused with YFP at their C-terminus; Km Cm | (57) |
| pRHTlp1 | pRH005 containing a tlp1 promoter region and ORF; Km Cm | (25) |
| pRHTlp4a | pRH005 containing a tlp4a promoter region and ORF; Km Cm | This work |
| pUT18 | Derivative of pUC19 plasmid encoding T18 of CyA, Cb | (58) |
| pKNT25 | Derivative of pSU40 plasmid encoding T25 of CyA, Km | (58) |
| pUT18C-zip | a derivative of pUT18C in which the leucine zipper of GCN4 is genetically fused in frame to the T18 fragment, Cb | (58) |
| pKT25-zip | a derivative of pKT25 in which the leucine zipper of GCN4 is genetically fused in frame to the T25 fragment, Km | (58) |
| pUT18cheA4 | pUT18 containing cheA4, Cb | This work |
| pUT18cheW4 | pUT18 containing cheW4, Cb | This work |
| pUT18tlp1 | pUT18 containing tlp1, Cb | This work |
| pUT18tlp4a | pUT18 containing tlp4a, Cb | This work |
| pUT18tlp4S | pUT18 containing truncated tlp4a, Cb | This work |
| pUT18cheA1 | pUT18 containing cheA1, Cb | This work |
| pUT18cheW1 | pUT18 containing cheW1, Cb | This work |
| pKNT25cheA4 | pKNT25 containing cheA4, Km | This work |
| pKNT25cheW4 | pKNT25 containing cheW4, Km | This work |
| pKNT25tlp1 | pKNT25 containing tlp1, Km | This work |
| pKNT25tlp4a | pKNT25 containing tlp4a, Km | This work |
| pKNT25cheA1 | pKNT25 containing cheA1, Km | This work |
| pKNT25cheW1 | pKNT25 containing cheW1, Km | This work |
| prh CheA1∆TMX | pRH005 vector containing CheA1∆TMX | (28) |
| prhCheA4 | pRH005 vector containing CheA1∆TMX | This work |
| prhTlp1 | pRH005 vector containing Tlp1 | This work |
| prhTlp4a | pRH005 vector containing Tlp4a | This work |
| pKNOCK-Tc | Suicide vector for gene knock out using insertion mutageneis; Tet | Addgene |
| pKNOCK_CheA2 | pKNOCK containing part of CheA2 for insertional mutagenesis | This work |

Antibiotics used: Km – kanamycin (50 µg/mL or 30 µg/mL), Cm- chloramphenicol (34 µg/mL), Gm – gentamycin (20 µg/mL), Cb – carbenicillin (50 µg/mL), Tet – tetracycline (10 µg/mL).
